# Combined effects of landscape fragmentation and sampling frequency of movement data on the assessment of landscape connectivity

**DOI:** 10.1186/s40462-024-00492-8

**Published:** 2024-09-09

**Authors:** Marie-Caroline Prima, Mathieu Garel, Pascal Marchand, James Redcliffe, Luca Börger, Florian Barnier

**Affiliations:** 1 PatriNat (OFB - MNHN), 75005 Paris, France; 2 Office Français de la Biodiversité, Direction de la Recherche et de l’Appui Scientifique, Service Anthropisation et Fonctionnement des Ecosystèmes Terrestres, 38610 Gières, France; 3 Office Français de la Biodiversité, Direction de la Recherche et de l’Appui Scientifique, Service Anthropisation et Fonctionnement des Ecosystèmes Terrestres, 34990 Juvignac, France; 4 Department of Biosciences, Swansea University, Swansea, SA15HF UK; 5 Centre for Biomathematics, Swansea University, Swansea, SA15HF UK; 6 PatriNat (OFB - MNHN), 91800 Brunoy, France

**Keywords:** Alpine ibex, Dead-reckoning, Distance-based networks, Landscape fragmentation, Minimum planar graph, Movement behaviour, Sampling frequency, Scale-free, Small-world, Spatial network theory

## Abstract

**Background:**

Network theory is largely applied in real-world systems to assess landscape connectivity using empirical or theoretical networks. Empirical networks are usually built from discontinuous individual movement trajectories without knowing the effect of relocation frequency on the assessment of landscape connectivity while theoretical networks generally rely on simple movement rules. We investigated the combined effects of relocation sampling frequency and landscape fragmentation on the assessment of landscape connectivity using simulated trajectories and empirical high-resolution (1 Hz) trajectories of Alpine ibex (*Capra ibex*). We also quantified the capacity of commonly used theoretical networks to accurately predict landscape connectivity from multiple movement processes.

**Methods:**

We simulated forager trajectories from continuous correlated biased random walks in simulated landscapes with three levels of landscape fragmentation. High-resolution ibex trajectories were reconstructed using GPS-enabled multi-sensor biologging data and the dead-reckoning technique. For both simulated and empirical trajectories, we generated spatial networks from regularly resampled trajectories and assessed changes in their topology and information loss depending on the resampling frequency and landscape fragmentation. We finally built commonly used theoretical networks in the same landscapes and compared their predictions to actual connectivity.

**Results:**

We demonstrated that an accurate assessment of landscape connectivity can be severely hampered (e.g., up to 66% of undetected visited patches and 29% of spurious links) when the relocation frequency is too coarse compared to the temporal dynamics of animal movement. However, the level of landscape fragmentation and underlying movement processes can both mitigate the effect of relocation sampling frequency. We also showed that network topologies emerging from different movement behaviours and a wide range of landscape fragmentation were complex, and that commonly used theoretical networks accurately predicted only 30–50% of landscape connectivity in such environments.

**Conclusions:**

Very high-resolution trajectories were generally necessary to accurately identify complex network topologies and avoid the generation of spurious information on landscape connectivity. New technologies providing such high-resolution datasets over long periods should thus grow in the movement ecology sphere. In addition, commonly used theoretical models should be applied with caution to the study of landscape connectivity in real-world systems as they did not perform well as predictive tools.

**Supplementary Information:**

The online version contains supplementary material available at 10.1186/s40462-024-00492-8.

## Background

Studying landscape connectivity is crucial for better understanding many ecological processes including animal foraging behaviour [1], population distribution [2, 3], gene or pathogen flow [4], migration behaviour [5], species interactions [6, 7], or metapopulation persistence [8]. Analysis of landscape connectivity can also be useful to identify critical areas favouring movements [9–11] or to evaluate and compare the effect of land planning actions (e.g., site restoration) [12, 13]. Consequently, an accurate assessment and prediction of landscape connectivity is needed to avoid spurious understanding of multiple theoretical and applied landscape connectivity-related processes.

Network (or graph) theory has been largely used in multiple taxa to model and predict connectivity in fragmented landscapes [14–18]. In such landscapes, habitat patches are represented using nodes while observed or predicted movements among patches are represented using links [19]. Links can either mirror the Euclidean distance between patches (i.e., straight lines) [20] or account for matrix composition among patches (i.e., least cost path) [6]. The popularity of spatial networks relies on their easy implementation and the large amount of connectivity indices that can be derived from a network providing quantitative information on landscape connectivity at different levels (i.e., node, group of nodes or network level) [21–23]. Their adequatedness in real landscapes for correctly inferring ecological processes is, however, less immediately clear.

**Fig. 1:**
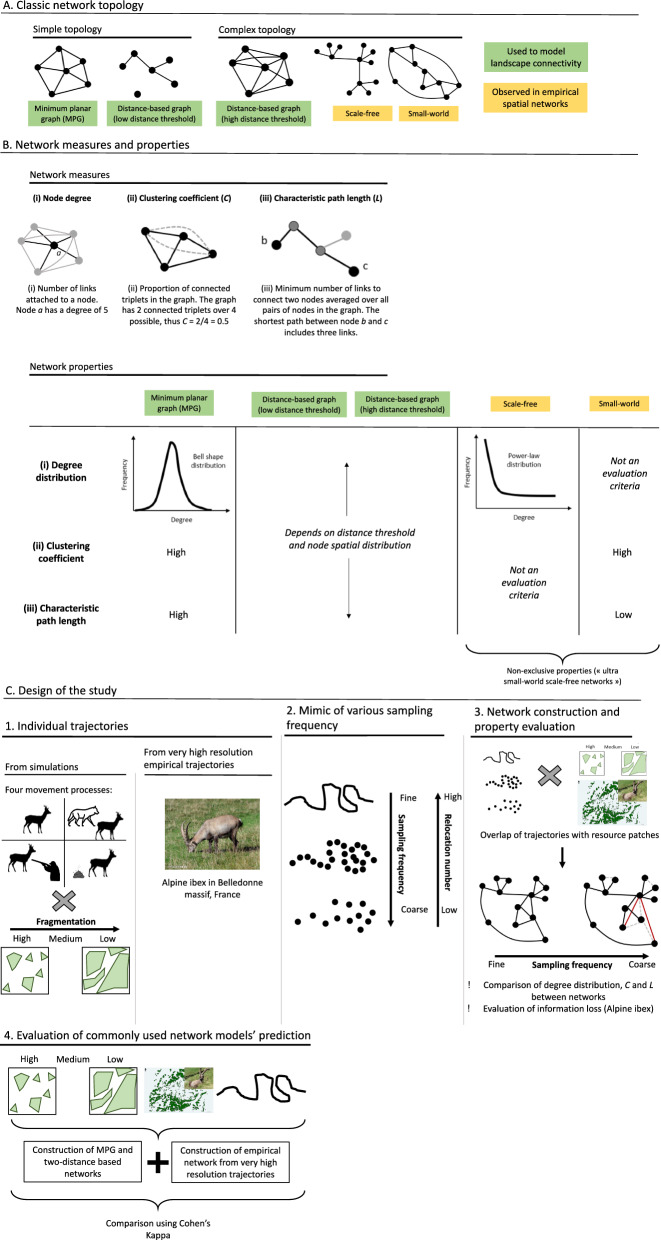
General description of network topologies and design of the study. Ibex photo ©: Franck Merlier

A landscape can be described using different network topologies including simple planar graphs (e.g., minimum spanning tree or minimum planar graph, MPG) [24] and more complex ones (e.g., scale-free or small-world networks) [25]. This result in different patterns of connectivity within the graph (Fig. 1A) [26]. For example, a MPG assumes that individuals move in a stepping stone fashion among resource patches, such that links in a MPG never cross (i.e., no shortcut). By contrast, in a scale-free network, few nodes (thereafter called hubs) are highly connected to the rest of the patches (thereafter called peripheral nodes) that have few connections [27]. Consequently, a scale-free network is highly sensitive to the removal of hubs but resistant to random removal of nodes, whereas a MPG shows similar effects of a disturbance independently of its spatial location [3, 28]. Network topology also has implications for processes occurring in spatial networks. Indeed, a disease would not spread much if it appears in a peripheral node while it would spread faster and to a larger extent if appearing in a hub or in a network having shortcuts among patches (such as in small-world networks) [20]. An accurate representation of actual landscape connectivity is thus of paramount importance to correctly identify network topology and, consequently, to understand and predict many landscape connectivity-related processes.

Application of spatial network theory to real-world landscapes generally relies on the modelling of either theoretical or empirical networks. Theoretical networks commonly include a MPG built from the distribution of resource patches [6, 15, 29], or a distance-based network built from both distribution of resource patches and empirically-estimated or expert-based dispersal distances [4, 9, 30]. Empirical graphs are generated by overlapping trajectories of remote-tracked individuals with resource patches to identify network links [17, 18, 31]. With the development of Global Positioning System (GPS) technology, many free-ranging species are now tracked over long periods of time [32, 33]. However, the weight constraints of collars fitted to animals limit battery capacities, generally resulting in a trade-off between relocation frequency and tracking duration depending on scientific questions [34]. For example, empirical networks have been built from GPS relocations collected every hour in plains bison (*Bison bison bison*) [3] or in African elephants (*Loxodonta africana*) [17], every two hours in woodland caribou (*Rangifer tarandus caribou*) [35], every four hours in brown bear (*Ursus arctos*) [36] or even every 24 h in several species of sea turtle [18]. However, the potential effect of relocation frequency on observed patterns of connectivity remains unclear. More generally, if the temporal dynamics of animal movement is too rapid compared to relocation frequency, detected patterns of landscape connectivity using networks might not reflect actual species movement behaviour. Indeed, discontinuous trajectories like the one generated from GPS monitoring could result in inaccurate estimations of inter-patch movements and, for example, the detection of artificial shortcuts due to missed stepping stone-like movements. In other words, an individual could stay 30 min in a feeding patch, then move to another feeding patch by making a quick stop along the way in a patch to drink but that series of inter-patch movements would not be detected if the individual relocation frequency is every hour or two hours. Consequently, relocation frequency could impact the assessment of connectivity in the sense that networks are perceived as more complex, while being actually simpler (e.g., artificial shortcuts could generate a small-world topology). On one hand, if networks are actually simpler than detected, it assures the relevant use of simple theoretical networks to predict landscape connectivity (i.e., such as the MPG). On the other hand, if networks are actually complex, there is an urgent need to develop theoretical network models that fit with complex functional patterns of connectivity.

In patchy landscapes, the level of habitat fragmentation could also impact observed patterns of landscape connectivity from remote-tracked individuals. Indeed, habitats show different levels of fragmentation starting from simple patch dissection to patch attrition with intermediate patterns such as patch shrinkage [37], resulting in habitat patches having different size, organisation and composition [38]. While patches can provide different resources to animals (e.g., food, shelter), their use (e.g., residency time, number of visits) can vary according to patch characteristics [3, 6, 35, 39]. For example, plains bison and woodland caribou were shown to stay longer in larger and highly profitable patches [35, 40]. Such variations in residency time within habitat patches could result in missed inter-patch movements (e.g., stepping-stones), notably when the sampling schedule is too coarse compared to the temporal dynamic of animal movement [41]. While the interplay between landscape fragmentation and relocation frequency could have noteworthy effects on the assessment of landscape connectivity using networks, it has yet to be appraised.

Finally, individuals could also adjust their movements as a response to space use of congeners, competitors or predators [42–45], such that landscape connectivity could result in different patterns according to the movement process at stake. For example, individuals can have limited access to the landscape when conspecifics are territorial and consequently have to restrain their movement to their own territory [46]. Similarly, space use and movements can be modified by preys in response to a threat (e.g., predator or human presence) [45, 47]. Consequently, we could expect network topology to be impacted by movement processes and, if so, these movement processes should be accounted for when building theoretical network models to predict connectivity in heterogeneous landscapes.

In this study, we address these outstanding methodological questions by investigating the combined effects of relocation sampling frequency and landscape fragmentation on the assessment of landscape connectivity from networks using (i) simulated trajectories accounting for four movement processes (foraging, foraging + avoidance of an elusive predator, foraging + avoidance of a stalking predator, foraging and territoriality) in simulated habitats with variable levels of fragmentation and (ii) empirical high-resolution (1 Hz) trajectories of Alpine ibex (*Capra ibex*) tracked in the northern French Alps (Belledonne massif) (Fig. 1C- panels 1-3). For both simulated and empirical trajectories, we also compared predictions from commonly used theoretical networks (i.e., MPG and distance-based graphs) to actual connectivity obtained from high-resolution data (Fig. 1C- panel 4).

## Methods

### Simulation study

#### Modelling of movement processes

We used a recently developed modelling framework to simulate individual trajectories in fragmented landscapes from continuous correlated biased random walks [48–50]. This framework simulates an animal grazing across stationary resources that deplete and regenerate, based on three processes: consumption and regeneration of resources, resource memory of the grazer, and state-specific biased correlated movement process [48]. The framework can also accommodate predator avoidance during movements by simulating predator appearance in the landscape and a resulting fleeing-behaviour by the grazer that memorizes an encounter location and avoids it for a parameterized period of time [49]. The model can finally include multiple territorial foragers that regularly scent-mark their territory and avoid scent-marks of conspecifics [50]. Detailed descriptions of foraging, predator avoidance and territoriality models are available in [48, 49] and [50], respectively, such that we only provide a brief description of each modelled process in Appendix S1.

#### Modelling of landscape fragmentation

We generated three types of landscapes of 50 × 50 cells with different levels of fragmentation using an exponential variogram model. This model was characterized by four parameters including the nugget (i.e., variogram’s intercept), the sill (i.e., variogram’s asymptote), the range (*r*, i.e., distance beyond which variables are no longer autocorrelated) and the trend (*t*, i.e., average predicted value over the landscape). Constant parameters of the model were: nugget = 0 and sill = 1. We set range and trend coefficients to obtain three levels of fragmentation: low (*r* = 5, *t* = 0.3), intermediate (*r* = 2, *t* = − 0.5) and high (*r* = 2, *t* = − 1.5) [51, 52]. We produced 100 landscapes for each set of parameters to account for stochasticity. Following [48], negative values were truncated to 0 and landscapes were normalized to sum to one. Thus, cells with positive values reflected resources of gradual quality while null values corresponded to non-resource cells. We used the R package *gstat* (v2.1-1) to produce the landscapes [54]. We then calculated four indices of landscape fragmentation for each simulated landscape to make sure fragmentation characteristics changed between the different landscapes. To do so, we first truncated cells with positive values to one to delimit resource patches. We then calculated the aggregation index (i.e., the ratio between the amounts of realized shared edges and maximum possible shared edges, range: [0–100]), the patch cohesion index (i.e., equivalent of ratio of patch perimeters over patch areas, range: [0–100]), the division index (i.e., the probability that two randomly selected cells are not located in the same patch, range: [0–1]) and the proportion of cells occupied by resource patches (range: [0–100]) [37]. We selected those indices because they range between 0 and 1 such that they are comparable between different landscapes. We used the R package *landscapemetrics* to calculate the fragmentation indices [55].

#### Model runs and construction of spatial networks

We ran each model (F: Foraging-only, F + Pe: Foraging and avoidance of an elusive predator, F + Ps: Foraging and avoidance of a stalking predator, F + T: Foraging and territoriality) in each simulated landscape resulting in 1200 simulations (100 landscapes of each type × 4 movement processes × 3 levels of landscape fragmentation) (Fig. 1C- panel 1). Continuous time models were implemented in Java, with time discretized with small regular intervals Δt approximating dt (available code at https://zenodo.org/records/6104214). For each simulation, we ran the model during 10 000 timesteps. As for the F + T model, territories have to emerge first, we ran 20 000 time steps and discarded the first 10 000 to only keep movement behaviour when territories were in place [50]. Model parameters for each simulated process were initialized based on [48–50], and are reported in Table S1.

All of the subsequent analyses were performed using the R software (v4.3.0, [53]). We then built a network for each complete simulated trajectory (i.e., 1200 networks). To do so, we identified all movements that occurred between two resource patches and used unique inter-patch movements to build the network (i.e., unweighted and undirectional network) (Fig. 1C- panels 2-3). Then, we regularly resampled each trajectory at various frequencies to mimic discontinuous trajectories like the one generated from GPS monitoring. Notably, we drew one location out of two, one out of three, etc.…, until one location out of fifty, resulting in 4999, 3333, etc.…, down to 199 movement steps, respectively, for each trajectory (Fig. 1C- panel 2). We then built the spatial networks using these resampled trajectories (Fig. 1C- panel 3).

**Fig. 2:**
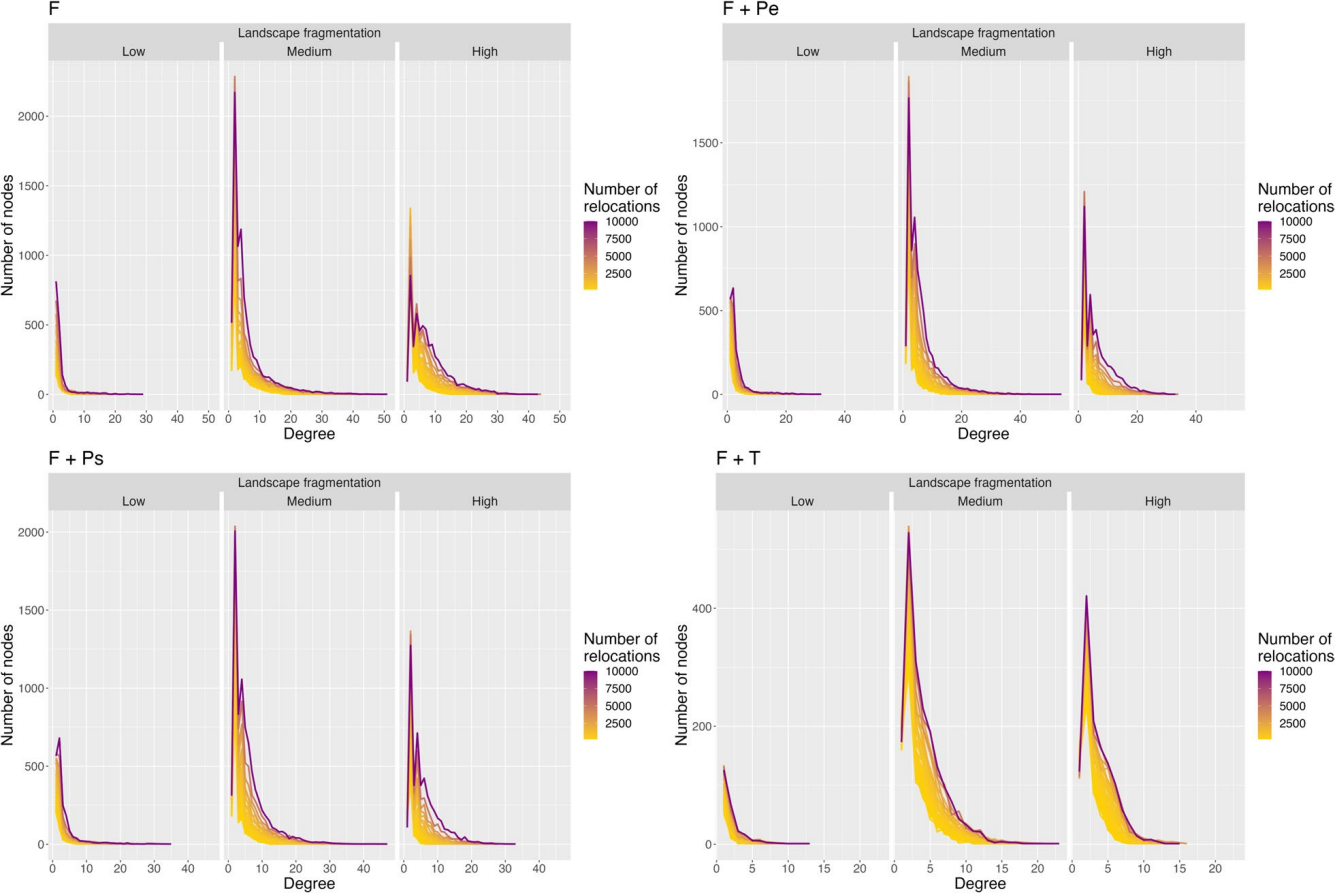
Degree distributions of simulated networks in three types of landscape fragmentation from four movement processes. F: Foraging, F + Pe: Foraging and avoidance of an elusive predator, F + Ps: Foraging and avoidance of a stalking predator, F + T: Foraging and territoriality. All distributions decayed as a power function with scale parameter being higher than 1 (Table S3)

### Evaluation of simulated spatial network

#### Effects of landscape fragmentation and sampling frequency on network topology

Multiple network metrics are available to describe landscape connectivity and to distinguish between network types [22]. Here, we focused on the degree distribution (i.e., the distribution of the number of links that each node has), the global clustering coefficient *C* (i.e., the number of connected triplets of nodes over the total number of triplets in the graph) and the characteristic path length *L* (i.e., the number of links in the shortest path between two nodes, averaged over all pairs of nodes) (Fig. 1B). These metrics allow evaluating whether a network topology is complex and similar to classic complex networks such as scale-free or small-world networks (Fig. 1A) [25]. In a scale-free network, the degree distribution follows a power-law distribution with scale parameter $\alpha$ > 1 (i.e., the probability of a node to be connected to $k$ other nodes, $P\left(X=k\right)\propto {k}^{-\alpha }, \alpha >1, k > \text{ 0}$), reflecting the presence of hubs and peripheral nodes within the network [27, 56]. In a small-world network, many triplets of nodes are connected resulting in a high clustering coefficient and some links provide long range connection between distant nodes (e.g., shortcuts) leading to a short characteristic path length (i.e., fast traversability of the graph) [56, 57]. The ad hoc method to evaluate whether a network is small-world is to compare *C* and *L* to the clustering coefficient and characteristic path length of an associated random graph built from the same number of links and nodes than the one of interest [58, 59]. Notably, in a small-world network, the clustering coefficient is much larger than the clustering coefficient of its associated random graph and the characteristic path length is similar to the one of its associated random graph.

**Fig. 3:**
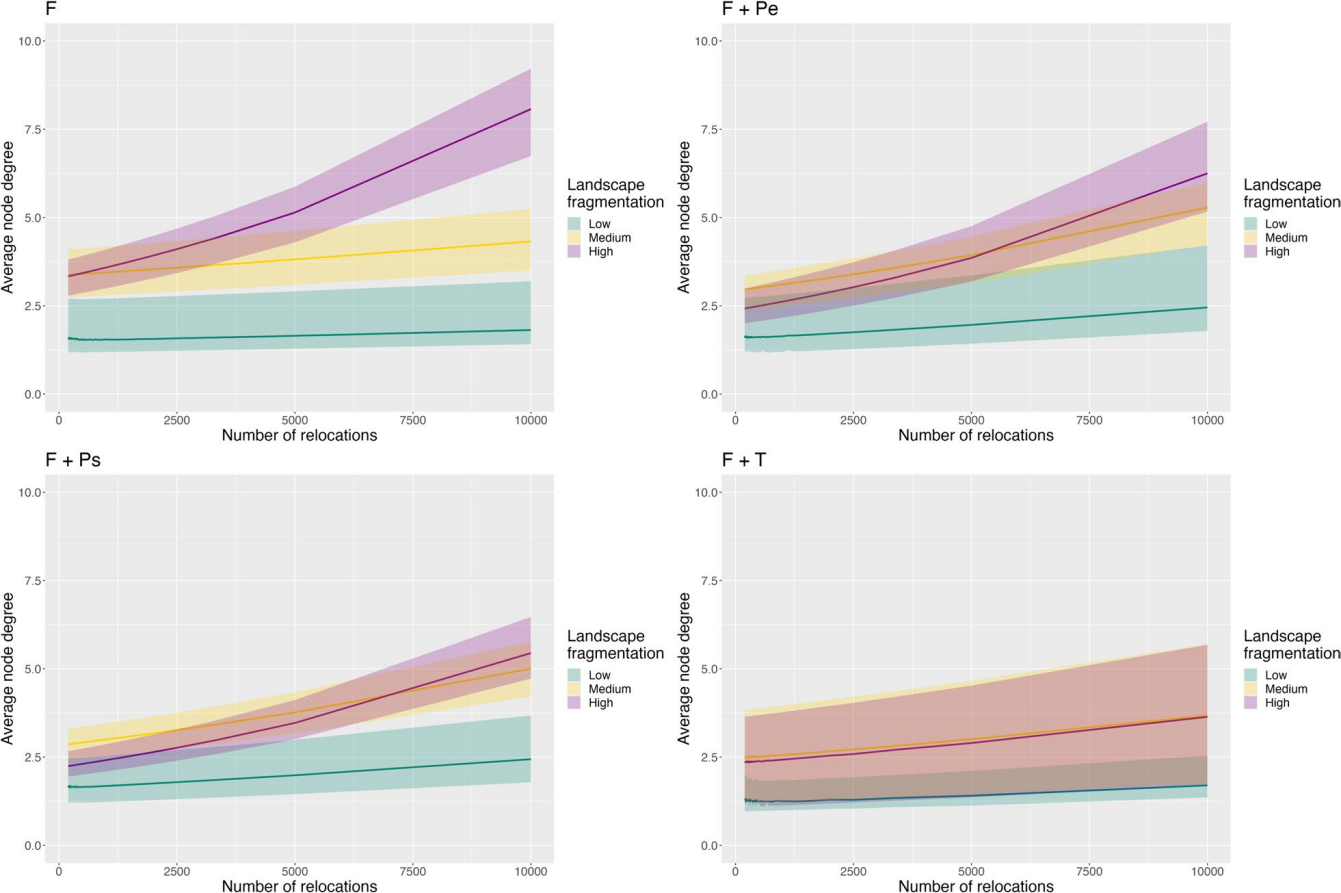
Estimates and their 95% confidence interval of average node degree of simulated networks. One model was run for each simulated movement process: F: Foraging, F + Pe: Foraging and avoidance of an elusive predator, F + Ps: Foraging and avoidance of a stalking predator, F + T: Foraging and territoriality. Each model included as covariates the level of landscape fragmentation, the number of relocations used to build the spatial network (correlated to the sampling frequency) and the interaction of both. Pseudo-R^2^ of the models were: F: 0.14; F + Pe: 0.18; F + Ps: 0.18; F + T: 0.26

To evaluate the effects of landscape fragmentation, sampling frequency and movement process driving forager behaviour on the network topology, we first extracted the node degree of all visited patches of each network. We then fitted a linear mixed-effects model to the log-transformed node degree as a function of landscape fragmentation, the number of relocations used to build the network (correlated to sampling frequency, Fig. 1C-2) and the interaction of both (Fig. 1C-3). We included a random effect of landscape ID to account for autocorrelation in the degrees of patches coming from the same landscape. We ran a model for each simulated movement process (i.e., F, F + Pe, F + Ps and F + T). We also computed network degree distribution for each level of landscape fragmentation and each sampling frequency and fitted the log-transformed frequency of node degrees to the log-transformed degrees using a linear model to estimate whether networks were scale-free (i.e., in such case estimated scale parameter should be higher than 1). We then extracted *C* and *L* of each network and built 100 random networks for each simulated network based on the same number of links and visited patches and compared their clustering coefficient (*C*_*R*_) and characteristic path length (*L*_*R*_) to *C* and *L* to assess whether the empirical network had the small-world property. More precisely, we followed [60] and calculated the ratio σ = (C/*C*_*R*_) / (L/*L*_*R*_) for each random graph meaning that we had one hundred calculation of σ for each simulated network. As these ratios should be higher than 1 if the simulated network is small-world [60], we defined a simulated network to be small-world if min(σ) > 1. Finally, we calculated the proportion of simulated networks being small-world for each simulated movement process as a function of landscape fragmentation and the number of relocations used to build the network. We used the R packages *igraph* (v1.4.3, [61]) to build the networks and calculate their connectivity indices, and *lme4* (v1.1–33, [62]) and *lmerTest* (v3.1–3, [63]) to fit the statistical models.

#### Adequacy with commonly used theoretical networks

We first built the minimum planar graph in each simulated landscape using the R package *grainscape* [53]. We also simulated two distance-based networks and a random graph in each landscape for each simulated movement process (Fig. 1C- panel 4). Notably, we built distance-based networks using two thresholds for link length: one corresponding to the median length (*L*_*med*_) of realized links obtained from unique inter-patch movements of the complete trajectory (DIST50), and one corresponding to the 95% quantile (*L*_*95%*_) of the distribution of realized link lengths (DIST95). Consequently, only patches that were closer than *L*_*med*_ or *L*_*95%*_ were thus connected in DIST50 and DIST95, respectively. For the random graph, we randomly assigned the same number of realized links among patches of the landscape. We then used the Cohen’s Kappa (*κ*) to assess the performance of theoretical spatial networks (MPG, DIST50, DIST95 and Random) to correctly predict connectivity emerging from different movement models in landscapes having various levels of fragmentation [64, 65]. We specifically calculated *κ* by comparing predicted links (0/1) of theoretical networks to realized links (0/1) from simulated movement processes in each landscape.

### Empirical study—movements of Alpine ibex in the Belledonne massif

#### Study area and Alpine ibex population monitoring

The empirical study took place in the Belledonne massif located in the northern French Alps (45° 13′ N, 6° 4′ E). The tagged Alpine ibex ranged over a 35km^2^ area at an altitude around 2100 m within the core of the massif. Ibex are notably adapted to movements in steep terrains, which provide them refuge from perceived predation risk, in particular the steepest rocky cliffs. However, cliffs generally provide few foraging resources, which are rather concentrated in Alpine grasslands [66, 67], such that ibex move frequently among various habitat types to get both food and cover. In total, 10 male ibex (between 7 and 12 years old) were monitored in 2017 using Lotek (3300S and Litetrack models) or Vectronic (Vertex Plus model) GPS collars. Relocation frequency varied between 1 location per hour to 1 location every 2 h and ibex were tracked most of the time nearly one year. Among these 10 ibex, 6 were additionally equipped with Daily Diary multi-sensor biologgers (Wildbyte Technologies 2020), which include tri-axial accelerometer and tri-axial magnetometer sensors recording at 25 Hz/8 Hz, respectively. Data from these biologgers were combined with GPS data collected every one or two hours to reconstruct the movement trajectory every second using the dead-reckoning technique (DR; hereafter GPS-enabled dead-reckoning) [68]. Given a starting point, DR uses the accelerometry and magnetometry data to calculate the speed and heading of an individual animal every second and thereby reconstruct the full high-frequency movement path [69]. To successfully use the DR method, in addition to correctly calibrating the sensors and correct for hard and soft iron bias (see [69]), it is important to recalibrate the reconstructed DR track as errors can accumulate and increase if not accounted for—this is typically done using GPS locations collected at a lower frequency, as detailed in [70]. We focused our analysis on the period for which we had very high-resolution trajectories for all 6 ibex (i.e., June) resulting in a total of 3412 GPS relocations (341 locations on average per individual, range: 204–395) from the 10 GPS-collared ibex and 18 070 914 DR relocations (3 011 819 locations on average per individual, range: 3 010 683–3 012 356) from the 6 ibex that were also equipped with biologgers. Variation in the number of relocations between ibex from GPS-enabled dead-reckoning came from a few missing relocation estimates but since this phenomenon was marginal (i.e., less than 0.5% of the dataset) and the time difference between two successive relocations never exceeded 4 s, we considered missing location to be the same as immediately preceding location.

We used slope and aspect layers provided by the R package *terra* to describe terrain characteristics in the ibex range at a resolution of 13 × 13 m. We also used description of habitats from the vegetation map provided by the *Conservatoire Botanique National Alpin* [71]. Notably, 19 habitats were identified within the ibex range from the vegetation map, which we reclassified into 8 classes including: closed forest, grassland, herbaceous—mineral, low ligneous, mineral, open forest, snow and other.

#### Identification of resource patches for Alpine ibex

To identify ibex resource patches, we first quantified their habitat selection using a resource selection function (RSF) and geolocations of the 10 GPS-collared ibex. In an RSF, characteristics of observed GPS points of an individual are compared to random locations [72]. For each observed location of Alpine ibex, we thus generated 5 random locations within the 99% contour of the utilization distribution (UD) of each individual. UD was estimated using a brownian bridge kernel calculated from the *kernelbb* function of the R package adehabitatHR with sig2 = 25 (i.e., GPS measurement error) and sig1 being estimated from the *liker* function of adehabitatHR [73–75]. Observed and random locations were then characterized by land cover types using the reclassified vegetation map (8 classes), the log-transformed distance to steep slope (i.e., slope > 40°, refuge effect) and the northness (i.e., sinus of aspect, potential thermal cover) [75]. We then used a generalized linear mixed-effects model with a binomial distribution to estimate RSF parameters. We included a random intercept for individual’s ID to control for the non-independence and the unbalanced number of observations per individual. Model robustness was assessed using k-fold cross validation [76]. More specifically, 80% of the individuals were randomly selected to fit the model. We then used the model to calculate predicted values for observed and random locations of the remaining 20% of individuals. Predicted values were divided into 10 equal bins such that observed and random locations obtained each a bin rank between 1 and 10. Bin rank (1–10) was then compared to the frequency of observed locations in each bin using Spearman’s rank correlation ($\overline{{r}_{s}}$). The procedure was repeated 100 times to obtain mean and range of ($\overline{{r}_{s}}$).

We then calculated from the fitted RSF a selection probability for each 13 × 13 m pixel of the study area and retained pixels with a probability above the 75% quantile of probability distribution. We then aggregated adjacent selected pixels to generate resource patches for ibex. We filtered patches on polygons larger than 2000 m^2^ to take into account the radius of GPS measurement error (i.e., π*25^2^ m^2^). Finally, once ibex resource patches were identified, we calculated the four indices of landscape fragmentation (i.e., the aggregation, patch cohesion and division indices, and the proportion of cells occupied by habitat patch) to identify to which fragmentation scenario from the simulated landscapes the ibex actual landscape was more similar. We used the R packages adehabitatHR (v0.4.21), sp (v1.6-1), stars (v0.6-1) and raster (v3.6-20) to perform all geographical information system work and lme4 (v1.1-33) and lmerTest (v3.1-3) to run the statistical analysis [52, 62, 63, 73, 77, 78].

#### Construction of Alpine ibex spatial network at various sampling designs

We used GPS-enabled dead-reckoning relocations obtained every second (1 Hz) for 6 ibex to first build a very high-resolution spatial network. As for the simulation study, we identified all movement steps that occurred between two resource patches and used unique inter-patch movements to build the network. We then regularly resampled each trajectory to mimic various sampling designs and built the resulting spatial networks. Notably, we resampled each trajectory by joining one location out of 30 (30 s), 60 (1 min), 300 (5 min), 600 (10 min), 1200 (20 min), 1800 (30 min), 3600 (1 h), 7200 (2 h) and 21,600 (6 h). We randomly selected the first relocation to start the resampling for each ibex and each sampling design to generate stochasticity. For example, when relocations were sampled every 30 s, the relocation to start with was randomly selected between the first 30 available relocations while for the sampling at 1 h, the relocation to start with was selected between the first 3600. In addition, for the 2 and 6 h sampling design, we also shifted the sampling time by one hour every day to have a complete sample of all possible hours. For example, for the 6 h sampling design, we sampled relocations day 1 at 00:00, 06:00, 12:00, 18:00 and 24:00, then day 2 at 01:00, 07:00, 13:00, 19:00, 01:00, then day 3 at 02:00, 08:00 and so on.

#### Evaluation of Alpine ibex spatial network

We performed the same analysis on ibex spatial networks as in the simulation study. We first evaluated the effect of sampling frequency on node degree by fitting a linear model to all log-transformed degrees as a function of the log-transformed number of relocations used to build the networks. We also assessed whether the networks had the scale-free and small-world properties by estimating the scale parameter of each degree distribution and by comparing their clustering coefficient and characteristic path length to the ones of random graphs, respectively (see section *Effects of landscape fragmentation and sampling frequency on network topology*). Secondly, we built the four commonly used theoretical spatial networks (i.e., MPG, DIST50, DIST95 and random graph) to predict connectivity among ibex resource patches. We restricted the predictions to patches that were encompassed within the 100% minimum convex polygon of the very high-resolution relocations (i.e., every second) of the 6 ibex. We then compared predictions of each theoretical network to the empirical observation of ibex spatial network derived from the very high-resolution trajectory (1 Hz), using Cohen’s Kappa.

In addition, we also calculated, for each network obtained from the resampled trajectories, the proportion of visited patches detected, the proportion of realized and spurious links detected. To do so, we used the spatial network built from the 1 Hz trajectory as the reference and extracted from it the lists of visited patches and realized links. We then evaluated whether these visited patches and links were found again in resampled networks. We defined spurious links as links that were observed in the resampled networks but missing from the reference network.

## Results

### Simulation study

#### Effects of landscape fragmentation and sampling frequency on simulated networks

Simulated landscapes showed various fragmentation characteristics according to the four indices of landscape fragmentation (described in Table S2). Each degree distribution calculated from 100 simulated networks decayed as a power function with scale parameter being greater than 1 (Table S3), indicating a strong heterogeneous pattern of connectivity among patches in all simulated networks. Few patches had many links (i.e., the hubs) and most of the remaining patches had few links, typical of scale-free networks (Figs. 2, S1 and S2). The scale-free property persisted even when sampling frequency decreased (i.e., fewer relocations) for all types of landscape fragmentation and all simulated movement processes (Fig. 2, Table S3). However, node degree depended on both sampling frequency and landscape fragmentation for all four simulated movement processes (Fig. 3). Node degree globally increased with the number of relocations but at a stronger rate in highly fragmented landscapes for the movement processes reflecting only foraging (i.e., F) or foraging and predator avoidance (i.e., F + Pe and F + Ps, Fig. 3), implying higher information loss on landscape connectivity in fragmented landscapes when sampling frequency gets coarser. This effect, however, dissipated when foragers had to constrain their movements within their own territory (Fig. 3, F + T).

**Fig. 4:**
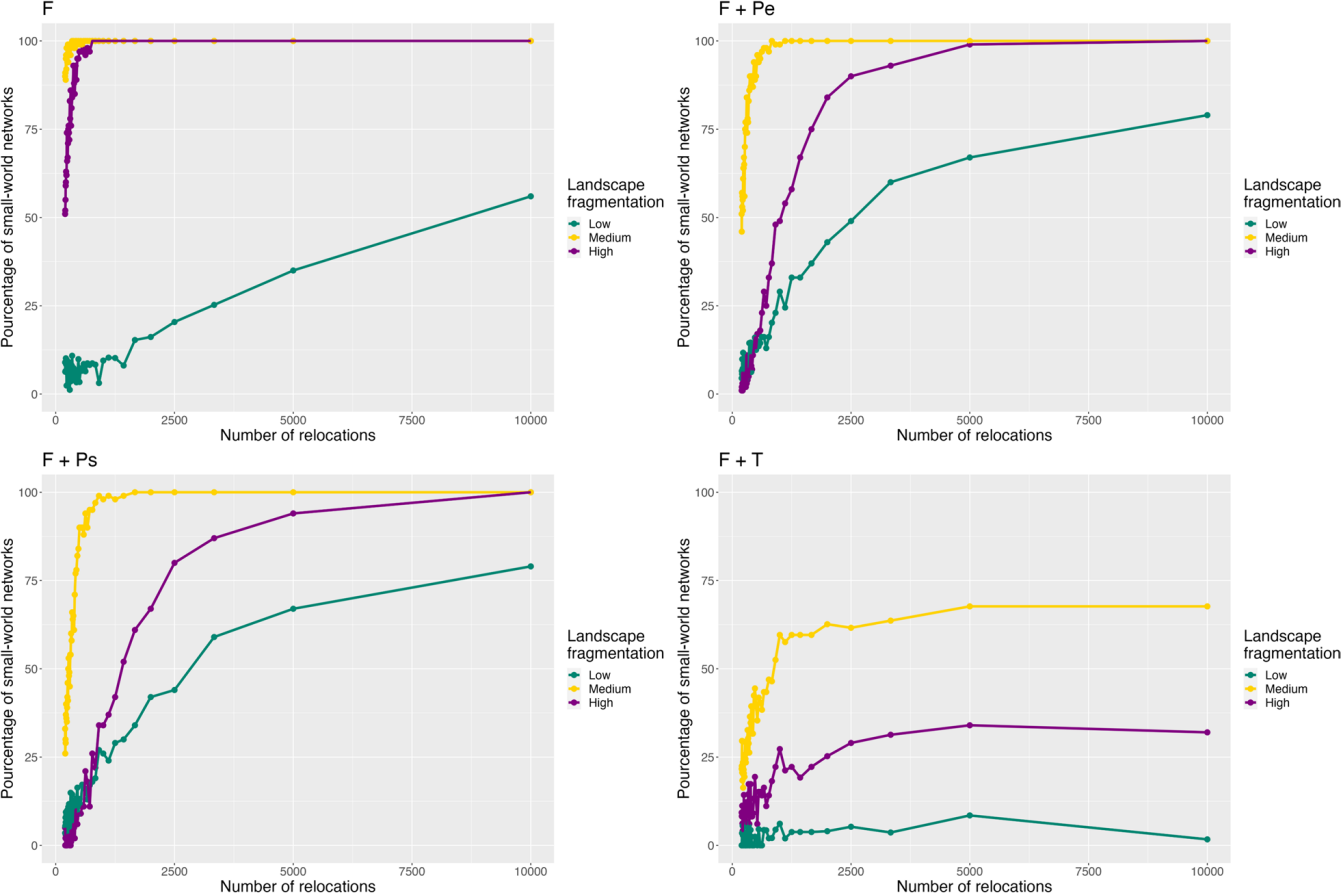
Pourcentage of simulated networks having the small-world property. The small-world property is defined as a relatively high clustering coefficient and a similar characteristic path length compared to a random graph. The number of relocations is correlated to the sampling frequency with higher number of relocations indicating fine sampling frequency. The combination of three levels of landscape fragmentation and four movement processes were simulated: F: Foraging, F + Pe: Foraging and avoidance of an elusive predator, F + Ps: Foraging and avoidance of a stalking predator, F + T: Foraging and territoriality

The emergence of the small-world property was linked to both the simulated movement process and the level of landscape fragmentation (Fig. 4). Indeed, from the full trajectory (i.e., 10,000 relocations), all simulated networks were small-world in highly and medium fragmented landscapes for the movement processes reflecting only foraging (i.e., F) or foraging and predator avoidance (i.e., F + Pe and F + Ps, Fig. 4). This proportion decreased though when landscapes had low levels of fragmentation or when territoriality was at stake in the movement process (F + T, Fig. 4). In addition, a decrease in the number of relocations generally led to a decrease in the proportion of small-world networks at a rate that depended on both the movement process and the fragmentation level (Fig. 4), meaning that a coarser sampling frequency impeded the detection of complex network topologies.

#### Adequacy with commonly used theoretical spatial networks

Commonly used theoretical networks showed various levels of accuracy to predict connectivity emerging from different movement processes in landscapes having various levels of fragmentation (Table 1) and never predicted correctly more than half of the landscape connectivity. Indeed, Cohen’s Kappa varied between 0 and 0.49 with lowest values for the random graph and highest values for the DIST50 in all four simulated movement processes and all levels of landscape fragmentation. The accuracy provided by the MPG was quite similar to the one of DIST50 although the DIST50 outperformed the MPG when landscape fragmentation was low and the foraging or foraging and territoriality processes were modelled, or when landscape fragmentation was high and the foraging process was simulated (Table 1). Note that the accuracy of both DIST50 and MPG dropped around 0.25 in highly fragmented landscape when foraging and predator avoidance or foraging and territoriality were simulated (Table 1).

**Table 1:** Adequacy (Cohen’s Kappa) between theoretical spatial networks and simulated networks from four movement processes

	F			F + Pe			F + Ps			F + T		
	Low	Med	High	Low	Med	High	Low	Med	High	Low	Med	High
Dist50	0.46	0.42	0.40	0.49	0.42	0.24	0.48	0.41	0.24	0.43	0.42	0.26
Dist95	0.30	0.16	0.13	0.33	0.16	0.06	0.28	0.14	0.05	0.41	0.29	0.09
MPG	0.39	0.41	0.31	0.48	0.42	0.23	0.47	0.41	0.24	0.31	0.42	0.26
Rand	0.01	− 0.00	0.01	0.00	− 0.00	0.00	0.00	0.00	− 0.00	0.00	0.01	0.03

The Cohen’s Kappa is calculated from the comparison of predicted links from four theoretical spatial networks and observed links obtained by simulation of individual trajectories from different movement processes in landscapes having various levels of fragmentation (low, medium (med) or high). Simulated movement processes: F: Foraging, F + Pe: Foraging and avoidance of an elusive predator, F + Ps: Foraging and avoidance of a stalking predator, F + T: Foraging and territoriality. Theoretical networks: DIST50: distance-based network where patches closer than L_50%_ are connected, DIST95: distance-based network where patches closer than L_95%_ are connected, MPG: minimum planar graph, Rand: random network where random patches are connected. L_50%_ is the median length of realized links obtained from unique inter-patch movements of the complete simulated trajectory and L_95%_ is the 95% quantile of the distribution of realized link lengths. Kappa can vary between − 1 and 1 with closer values to 1 indicating perfect match

### Case study

#### Identification of resource patches for Alpine ibex

Habitat selection by male ibex in June depended on both the distance to steep slopes and land cover class (Table S4). Males selected notably low levels of ligneous cover and avoided mineral and snow covered areas when compared to grasslands (i.e., reference habitat). Besides, they selected cover that was close to steep slopes (Table S4). Ibex resource patches were highly aggregated as the aggregation index was 78.4 and the patch cohesion index reached 96.2. However, the division index was equal to 0.90 indicating that despite being aggregated, patches were also highly divided in the landscape. The proportion of cells occupied by habitat patches totalled 23.8%. Consequently, the level of fragmentation in ibex landscape was relatively similar to the one of landscapes defined as medium or low fragmention in our simulations, according to these four indices of landscape fragmentation.

#### Evaluation of Alpine ibex spatial network

**Fig. 5:**
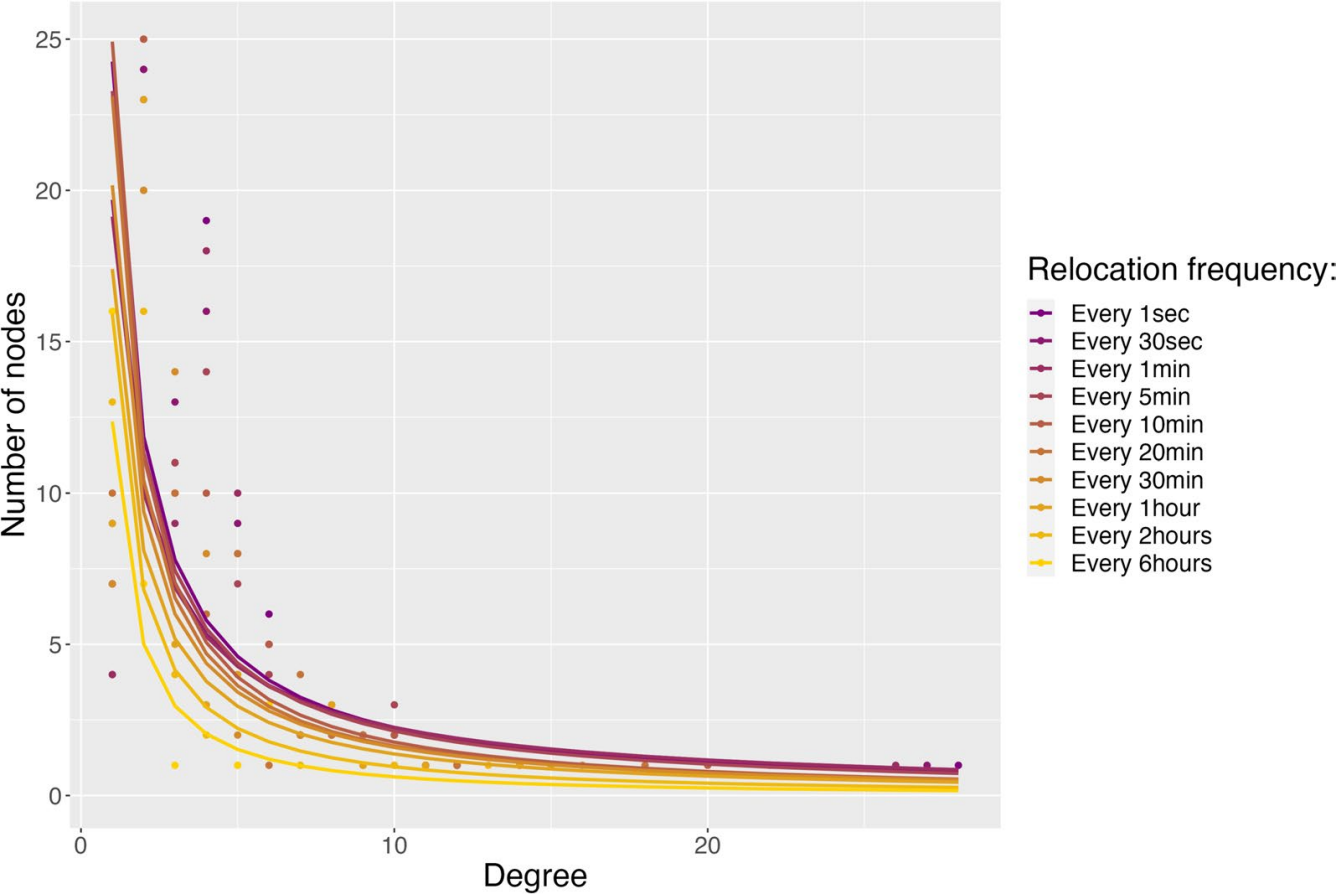
Degree distributions of Alpine ibex spatial networks in Belledonne massif (French Alps) in June 2017. Colors indicate the relocation frequency used to build individual trajectories that generated the spatial network. Lines indicate estimated degree distribution from a power law ajusted to the observed degree distribution (points)

Similarly to the simulation results, topology of ibex spatial network was impacted by sampling frequency as node degree increased significantly with the number of relocations used to build individual trajectory ($\text{log}\left(D\right)=0.043 \left(\pm 0.010\right)*\text{log}\left(N\right), p<0.001, {R}^{2}=0.03$, Fig. 5). The amount of variance explained (${R}^{2}$) was however quite low indicating that other factors should impact patch connectivity more strongly. In addition, most ibex networks were scale-free according to the estimation of scale parameter (i.e., $\widehat{\alpha }>1$, range for scale-free networks [min–max]: 1.03–1.30, ${R}^{2}$ of fitted models [min–max]: 0.47–0.75). However, two networks (i.e., sampling relocations every 30 s and 1 min) did not show scale-free properties (i.e., ${\widehat{\alpha }}_{every 30 sec.}$ = 0.94, R^2^ = 0.49 and ${\widehat{\alpha }}_{every 1 min.}$= 0.93, R^2^ = 0.47) even though their scale parameter were both very close to one. Furthermore, most ibex networks had the small-world property according to the comparison of their clustering coefficient and characteristic path length to the one of random graphs (Table 2). This property was however not detected when sampling frequency was too coarse (i.e., relocations every 6 h, Table 2).

**Table 2:** Properties of Alpine ibex spatial networks in the Belledonne massif (French Alps) in June 2017

Sampling frequency	Num. of locations	Num. of visited patches	Num. of links	$\widehat{\alpha }$[*R*^*2*^]	Scale-free	*C*	*L*	Small-world
Every 1 s	18 070 914	83	170	1.03 [0.60]	Yes	0.42	4.84	Yes
Every 30 s	604 720	80	166	0.94 [0.49]	No	0.44	4.84	Yes
Every 1 min	302 380	80	167	0.93 [0.47]	No	0.44	4.86	Yes
Every 5 min	60 480	76	154	1.03 [0.65]	Yes	0.39	4.75	Yes
Every 10 min	30 240	73	142	1.15 [0.75]	Yes	0.46	4.79	Yes
Every 20 min	15 120	67	133	1.15 [0.71]	Yes	0.42	4.79	Yes
Every 30 min	10 080	63	119	1.10 [0.68]	Yes	0.43	4.54	Yes
Every 1 h	5 040	55	100	1.10 [0.74]	Yes	0.49	3.75	Yes
Every 2 h	2 619	42	59	1.22 [0.75]	Yes	0.22	3.22	Yes
Every 6 h	1 005	28	28	1.30 [0.47]	Yes	0.11	3.71	No

Sampling frequency indicates the frequency at which we resampled the full individual trajectories (i.e., every 1s) to further build ibex spatial network. $\hat{\alpha }$ [R^2^]: scale parameter estimate of network degree distribution and its associated model’s R-squared, C: network clustering coefficient, L: network characteristic path length

**Fig. 6:**
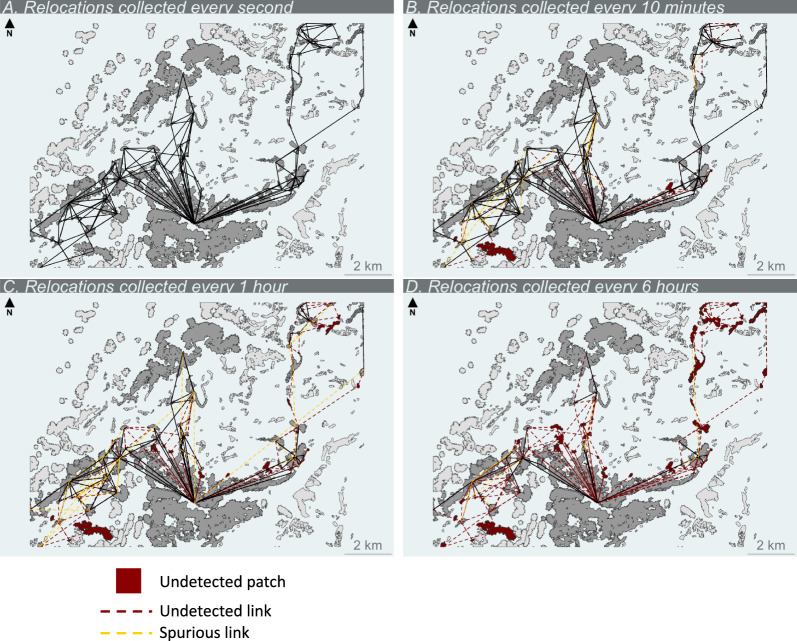
Alpine ibex spatial networks for three sampling schedules in Belledonne massif (French Alps) in June 2017. Resources patches are shown in light grey and black lines indicate inter-patch movements identified from ibex trajectories. Red patches and red dotted links are patches and links, respectively, that are detected from the full trajectory but not with the resampled trajectory. Yellow dotted links represent spurious links that were only detected with the resampled trajectory

**Fig. 7:**
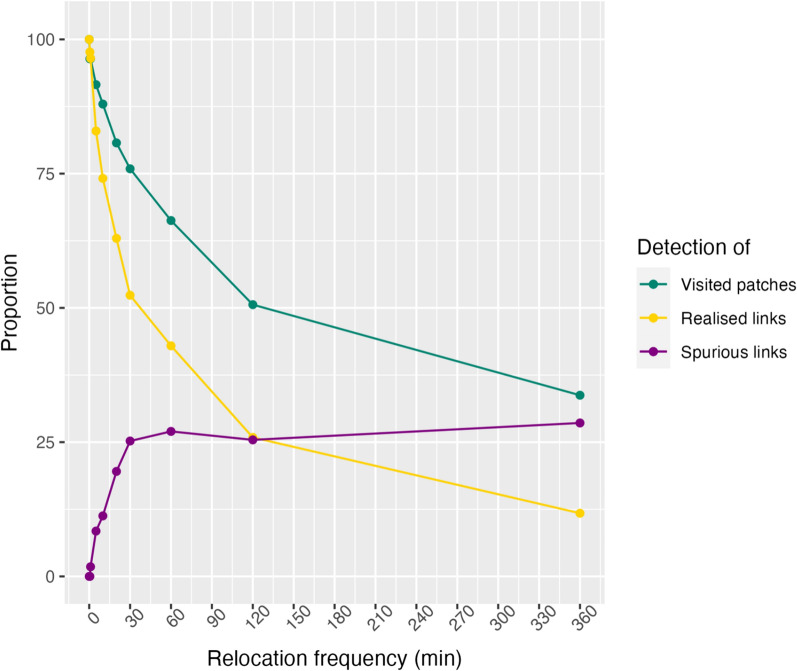
Effect of sampling frequency on information loss in Alpine ibex spatial networks. Proportion of visited patches detected (green), proportion of realized links detected (yellow) and proportion of spurious links detected (purple) in Alpine ibex spatial networks in Belledonne massif (French Alps) in June 2017 as a function of relocation sampling frequency

A strong loss of information on animal movement appeared quickly when sampling frequency became coarser (Figs. 6, 7). For example, when relocations were collected every 30 min, we detected 76% of visited patches and 52% of realized links and when relocations were collected every 2 h, these values dropped to 51% and 26%, respectively. This loss of information did not evolve linearly with sampling frequency as a significant amount of information was lost within the range of one relocation every 5 min to one relocation every 30 min (Fig. 7). Sampling frequency also impacted the detected pattern of connectivity as many identified links were actually spurious (Fig. 7). For example, 20% of detected links were spurious when relocations were sampled every 20 min and it raised to 27% when relocations were collected every hour (Fig. 7).

Commonly used theoretical networks showed relatively poor capacity to accurately predict landscape connectivity derived from our empirical dataset on Alpine ibex. Indeed, Cohen’s Kappa equalled 0.29, 0.10, 0.26 and 0.00 for the DIST50, DIST95, MPG and random graphs, respectively. Consequently, the DIST50 predicted more accurately inter-patch movements for ibex than the MPG, even though the difference is weak, but it only predicted successfully about one-third of landscape connectivity.

## Discussion

Using both simulated and empirical movement trajectories at very high-resolution of individuals showing different movement behaviours in landscapes with varying levels of fragmentation, we first demonstrated that an accurate assessment of landscape connectivity patterns can be hampered when the relocation sampling frequency is too coarse compared to the temporal dynamics of animal movement. However, we also revealed that the level of landscape fragmentation and the processes driving animal movement can both mitigate the effect of relocation sampling frequency on the detection of accurate landscape connectivity patterns. In addition, we showed that network topologies emerging from different movement behaviours and a wide range of landscape fragmentation were generally complex, and that commonly used theoretical networks, usually of simple topologies, did not perform well to accurately predict landscape connectivity in such environments. Finally, we also pinpointed that a significant amount of information on landscape connectivity is rapidly lost when sampling frequency of individual relocations becomes coarser.

Empirical spatial networks have been built from individual trajectories obtained at varying sampling frequencies without knowing so far the potential effect of trajectory resolution on the assessment of landscape connectivity using networks [6, 17, 18, 35, 36, 79, 80]. Here, we demonstrated that the sampling frequency of individual relocations can notably impede the detection of network small-world property but the strength of this effect was strongly linked to the level of landscape fragmentation. Indeed, a higher number of relocations (i.e., finer sampling frequency) was necessary to detect the small-world property of simulated networks in low fragmented than in highly or medium fragmented landscapes. The characteristics of small-world network result in many connected triplets of nodes (i.e., potential local functional use) with efficient movement within the network (i.e., through shortcuts) [57, 59]. Consequently, the missed detection of such patterns could result in inaccurate understanding of how landscapes are connected and for example, could lead to missed identification of patches or connections used for specific functional attributes (e.g., stopovers or stepping-stones) [31, 41, 81]. Similarly, we did not detect the small-world property of Alpine ibex empirical network when sampling frequency was too coarse (i.e., one location every 6 h). On the other hand, the scale-free property of simulated networks was always detected independently of the grain of relocation sampling frequency. However, the skewness of network degree distribution was impacted by both the level of landscape fragmentation and sampling frequency. Indeed, the local connectivity (i.e., node degree) was generally less heterogeneous (i.e., less skewed) in patchy landscapes when full trajectories were used to infer landscape connectivity but when sampling frequency became coarser, the skewness of degree distribution became stronger. These results indicate that commonly used discontinuous trajectories could lack robustness to infer landscape connectivity in real-world systems.

While habitat patch characteristics can potentially reduce the effect of sampling frequency on detected patterns of connectivity, the mitigation could yet be effective in opposite landscapes (i.e., high vs low fragmentation) depending on network topology (i.e., scale-free or small-world). Notably, in low fragmented landscapes, fine sampling frequency should be set to correctly detect small-world property while in highly fragmented landscapes, fine sampling frequency should be set to accurately assess scale-free property, according to our simulations. Many studies demonstrated that animals adjust their behaviour regarding patch selection and intensity of use as a response to patch environmental attributes [40, 82–85]. We indeed demonstrated that movements in landscape having different attributes can generate different connectivity patterns and that such patterns actually require fine sampling frequency to be correctly appraised. For example, we showed that about 50% of information on ibex movements in a low to medium fragmented environment was lost when ibex were relocated every 30 min and about 25% of their inferred movements were spurious with this sampling rate. While vertebrate movements are generally drawn from discontinuous individual trajectories collected at a coarser rate than one location per hour [18, 36, 80, 86, 87], our empirical study raised a warning on the use of such movement inference to the assessment of landscape connectivity from spatial networks.

The effect of relocation sampling frequency on detected patterns of landscape connectivity was also largely mitigated when simulated individuals were territorial in all types of fragmented landscapes. In the simulation, territorial individuals constrained their movements to their own territory due to the presence of conspecific scent-markings that were avoided [50]. Consequently, territorial individuals had access to a smaller part of the landscape than non-territorial individuals (i.e., F, F + Pe and F + Ps scenarios) such that movements within their territory were probably more redundant resulting in less information loss from coarser sampling frequencies. While our simulations provide new insights on the interplay between animal movement behaviour, landscape fragmentation and relocation sampling frequency on detected patterns of landscape connectivity, it has yet to be appraised using more empirical studies to provide additional support to our results. It could notably be helpful to further improve the design of studies aiming at evaluating landscape connectivity from empirical networks.

Most of our results, either from empirical or simulated cases, conclude that very high-resolution trajectories should be used to correctly infer patterns of landscape connectivity from empirical networks. New technological developments in animal tracking should help meeting this need. Indeed, a wide range of species, from small to large sizes, can now be tracked using GPS tags that collect more frequent data and over longer periods that in the past (e.g., by relying on solar energy) [88]. GPS relocations can be collected as frequently as every second, but generally at a cost of limited duration of tracking due to the high battery needs [89]. The application of dead-reckoning techniques to biologging and GPS data could also help collecting very high-resolution trajectories over longer periods [90]. DR relies on accelerometry and magnetometry data to calculate speed and heading along individual trajectories that can further be translated into very high-resolution coordinates (e.g., location every second, [68]). As battery constraints are less limited for magnetometers and accelerometers, sensor data can be collected much more frequently than solely GPS data and, when combined together to correct for magnetic drift, proved to be quite performant [68, 90]. In this study, Alpine ibex were equipped with tri-axial accelerometer and tri-axial magnetometer sensors recording at 25 Hz/ 8 Hz, respectively, and we combined those data with GPS data collected every two hours to produce locations every second [68]. DR has largely been used on domestic and aquatic wild species but its application remains rare for terrestrial wild species while it could provide detailed information about the movement paths of animals between GPS fixes and open new opportunities in movement ecology [91–95].

Spatial networks reflecting movements among resource patches have been identified as having complex topologies in a large body of litterature for various species [3, 31, 35, 79, 96]. We suggested that network topologies could be simpler than actually detected due to missed stepping-stone movements from coarse sampling frequency and consequently be more similar to simple topologies like the MPG or distance-based networks. Here, we refuted this hypothesis as all networks obtained from very high-resolution trajectories were actually complex (i.e., always scale-free and most of the time small-world). Observations of complex patterns of landscape connectivity could actually be expected as animal movement results from the complex interplay of animal state, navigation and motion capacity and external factors [43]. Consequently, commonly used theoretical networks did not succeed to accurately predict landscape connectivity in our simulated or empirical landscapes. These network models make general and relatively simple hypotheses on the drivers of animal movement among resource patches and are actually quite commonly used as decision-making tools [29, 97–99]. However, we demonstrated that they should be used with caution as they only succeeded to correctly predict between 30 and 50% of landscape connectivity when tested for various movement behaviours in different landscapes. Other tools such as individual based models (IBMs), have been combined with network theory to assess landscape connectivity [100, 101]. An IBM models individual movement in a landscape from parameterized rules of movements and consequently allows to generate mechanistic-based stochastic movement trajectories in heterogeneous environments [96, 102, 103]. Many IBMs have been developped to simulate dispersal behaviour in heterogeneous landscapes (i.e., individual based dispersal models [101]) notably because IBMs can address questions even when empirical knowledge is insufficient for linking individual-level processes to landscape-level patterns [104]. However, the application of IBM to the study of landscape connectivity using networks in the context of home-ranging behaviour (e.g., including recursive movements [105, 106]) remains underexploited and could cope with the urgent need to develop theoretical network models that fit with complex but more realistic functional patterns of connectivity [104].

## Conclusions

Animal behaviour, the level of landscape fragmentation and the frequency of individual relocations were shown to have an effect on the assessment of landscape connectivity using spatial networks. In general, very high-resolution trajectories (e.g., relocations collected every 1 min in Alpine ibex) were necessary to accurately identify complex network topologies and avoid the generation of spurious information on landscape connectivity. New technologies providing such high-resolution datasets over long periods, as for example dead-reckoning techniques, should thus grow in the movement ecology sphere to help unravelling many ecological questions and conservation challenges. In addition, commonly used theoretical models should be applied with caution to the study of landscape connectivity in real-world systems as they did not perform well as predictive tools. Instead, other modelling tools should be challenged to assess whether they can correctly generate more realistic functional patterns of landscape connectivity in actual environments.

## Supplementary Information

Supplementary material

## Data Availability

The datasets used and/or analysed during the current study are available from the corresponding author on reasonable request.

## Abbreviations

DIST50: Distance-based network where patches closer than the median realized link length are connected
DIST95: Distance-based network where patches closer than the 95% quantile of realized link length are connected
DR: Dead-reckoning
GPS: Global positioning system
IBM: Individual-based model
MPG: Minimum planar graph
