# Supplementary material for: Combined effects of landscape fragmentation and sampling frequency of movement data on the assessment of landscape connectivity
Source: Mov Ecol. 2024 Sep 9;12:63. doi: 10.1186/s40462-024-00492-8 (PMC11385819; doi:10.1186/s40462-024-00492-8)
Supplement: Supplementary file 1 — Supplementary material [file 40462_2024_492_MOESM1_ESM.docx]

**Supporting information for:** **Combined effects of landscape fragmentation and sampling frequency of movement data on the assessment of landscape connectivity**

Marie-Caroline Prima^a,b^, Mathieu Garel^b^, Pascal Marchand^c^, James Redcliffe^d^, Luca Börger^d,e^, Florian Barnier^f^

^a^PatriNat (OFB - MNHN), 75005 Paris, France;

^b^Office Français de la Biodiversité, Direction de la Recherche et de l’Appui Scientifique, Service Anthropisation et Fonctionnement des Ecosystèmes Terrestres, 38610 Gières, France ;

^c^Office Français de la Biodiversité, Direction de la Recherche et de l’Appui Scientifique, Service Anthropisation et Fonctionnement des Ecosystèmes Terrestres, 34990 Juvignac, France;

^d^Department of Biosciences, Swansea University, SA15HF Swansea, UK

^e^Centre for Biomathematics, Swansea University, SA15HF Swansea, UK

^f^PatriNat (OFB - MNHN), 91800 Brunoy, France;

Corresponding author: Marie-Caroline Prima^a,b^, [marie-caroline.prima@univ-grenoble-alpes.fr](mailto:marie-caroline.prima@univ-grenoble-alpes.fr)

**This pdf includes:**

- Appendix S1
- Tables S1 to S4
- Figures S1 to S3

**Appendix S1. Overview of the modelling framework used to simulate individual trajectories in heterogeneous landscapes.**

*Foraging model*

Resource consumption occurs at forager’s location but also more or less in the vicinity of the animal location and according to a defined consumption rate [1]. Resource regeneration occurs continuously throughout the landscape according to a defined regeneration rate and up to the initial value of resource abundance.

The memory map of the animal works as a combination of a long-term (attractive effect) and a short-term (repulsive effect) memory, each being regularly updated according to specific learning and decaying rates. As a result, the forager tends to avoid recently depleted patches and move towards known high quality patches.

The movement process of the animal is determined according to one of the two behavioural states it can be in: either feeding or searching. The individual switches from feeding mode to searching mode when its instantaneous rate of consumption drops below the average consumption rate, and inversely. In the feeding mode, the individual moves according to a continuous correlated random walk. In the searching mode, the individual moves according to a continuous biased correlated random walk, for which the bias is determined from the memory map weighted by a spatial kernel of distance with exponential distribution, such that the animal searches for known productive patches that are also close to its location. The autocorrelation in movement direction is stronger in the searching mode than in the feeding mode and the individual also moves faster when searching than feeding.

*Foraging and predator avoidance model*

In the model simulating predator avoidance by the forager, the animal consumes resources and moves among patches, and resources regeneratem using the same rules as presented above (see *Foraging model*). However, a predator can randomly appear in the landscape. It remains there for a period of time, and then disappears [2]. Both predator appearance location and persistence can be parameterized such that this location can be more or less correlated with forager resource distribution and the predator can stay for a shorter or longer time. A forager can detect predator presence according to a specific encounter radius and when it does, the animal moves directly away from the predator. The forager also memorizes encounter location and avoids this perceived risk of predation in its following searching moves by combining attraction towards known high-quality patches and repulsion of known risky places. As for the memory of resource patch quality, memory of predator encounter locations also decays with time.

*Foraging and territoriality model*

In the model simulating territoriality, several foragers consume resources and move among patches but each is constrained by territories of conspecifics [3]. Indeed, each individual scent-marks its territory at a specific rate and avoids scent-marking of conspecifics. Scents decay at a specific uniform rate meaning that individuals have to regularly mark their home-range to keep it as a territory. All conspecific scent-marks are integrated in the calculation of movement direction (repulsive effect decaying with distance to marks) during both feeding and searching moves. Other processes remain the same as for the foraging-only model (i.e., resource consumption and regeneration, resource memory).

**Cited litterature**

1. Bracis C, Gurarie E, Van Moorter B, Goodwin RA. Memory effects on movement behavior in animal foraging. PLoS One. 2015;10:e0136057.

2. Bracis C, Gurarie E, Rutter JD, Goodwin RA. Remembering the good and the bad: memory-based mediation of the food–safety trade-off in dynamic landscapes. Theor Ecol. 2018;11:305–19.

3. Theng M, Milleret C, Bracis C, Cassey P, Delean S. Confronting spatial capture–recapture models with realistic animal movement simulations. Ecology. 2022;e3676.

**Table S1.** Values and definition (from^a,b,c^) of model parameters used to simulate individual trajectory in fragmented landscape from four movement processes: F: Foraging, F+Pe: Foraging and avoidance of an elusive predator, F+Ps: Foraging and avoidance of a stalking predator, F+T: Foraging and territoriality.

| **Parameter** | **Definition** | **Process-specific values** | | | |
| --- | --- | --- | --- | --- | --- |
|  |  | **F** | **F+Pe** | **F+Ps** | **F+T** |
| **Simulations** |  |  |  |  |  |
| $\Delta t$ | Model time step | 1 | 1 | 1 | 1 |
| $T$ | Simulation length | 10 000 | 10 000 | 10 000 | 20 000 (first 10 000 discarded) |
| N | Number of foragers | 1 | 1 | 1 | 5 |
| **Consumption** |  |  |  |  |  |
| $\beta_{R}$ | Regeneration rate | 0.01 | 0.01 | 0.01 | 0.01 |
| $\beta_{C}$ | Consumption rate | 1 | 1 | 1 | 1 |
| $\gamma_{C}$ | Consumption spatial scale | 1 | 1 | 1 | 1 |
| **Resource memory^d^** |  |  |  |  |  |
| $\psi_{M}$ | Short-term memory factor | 2 | 2 | 2 | 2 |
| $\beta_{L},\beta_{S}$ | Learning rates | 1 | 1 | 1 | 1 |
| $\phi_{L},\phi_{S}$ | Decay rates | 0, 0.01 | 0, 0.01 | 0, 0.01 | 0, 0.01 |
| $\gamma_{L},\gamma_{S}$ | Learning spatial scales | 1 | 1 | 1 | 1 |
| **Movement^e^** |  |  |  |  |  |
| $\tau_{S},\tau_{F}$ | Autocorrelation time scales | 4, 2 | 4, 2 | 4, 2 | 4, 2 |
| $v_{S},v_{F}$ | Speeds | 6, 1 | 6, 1 | 6, 1 | 1, 0.15 |
| $\gamma_{Z}$ | Memory spatial scale | 10 | 10 | 10 | 10 |
| $\lambda$ | Mean time to update $\theta$ | 1 | 1 | 1 | 1 |
| **Predator behavior** |  | | | | |
|  | Total predation pressure | - | 5000 | 5000 | - |
| δ | Predator persistence | - | 10 | 150 | - |
| ρ | Predator spatial correlation | - | 0.1 | 0.8 | - |
| ε | Encounter radius | - | 5 | 5 | - |
| **Predation memory** |  | | | | |
| $\psi_{P}$ | Response strength | - | 1 | 100 | - |
| $\beta_{P}$ | Learning rate | - | 1 | 100 | - |
| $\phi_{P}$ | Decay rate | - | 0.1 | 0.001 | - |
| $\gamma_{P}$ | Learning spatial scale | - | 5 | 5 | - |
| $\gamma_{R}$ | Predation risk spatial scale | - | 10 | 10 | - |
| **Scent behavior** |  | | | | |
| $\beta_{D}$ | Deposition rate | - | - | - | 1 |
| $\gamma_{D}$ | Deposition spatial scale | - | - | - | 1 |
| $\phi_{D}$ | Decay rate | - | - | - | 0.0002 |
| $\gamma_{w}$ | Response spatial scale | - | - | - | 1.5 |
| $\psi_{D}$ | Response strength | - | - | - | 5 |

^a^Bracis, C., E. Gurarie, J. D. Rutter, and R. A. Goodwin. 2018. Remembering the good and the bad: memory-based mediation of the food–safety trade-off in dynamic landscapes. Theoretical Ecology 11:305–319.

^b^Bracis, C., E. Gurarie, B. Van Moorter, and R. A. Goodwin. 2015. Memory effects on movement behavior in animal foraging. Plos One 10:e0136057.

^c^Theng, M., C. Milleret, C. Bracis, P. Cassey, and S. Delean. 2022. Confronting spatial capture–recapture models with realistic animal movement simulations. Ecology n/a:e3676.

^d^L = long-term memory, S = short-term memory.

^e^S = searching, F = feedind.

**Table S2.** Mean and range (minimum-maximum) of the four indexes of landscape fragmentation for 100 simulated landscapes of each type of landscape patchiness. Range of indexes: aggregation index = [0 - 100] ; patch cohesion index = [0- 100] ; division index = [0 - 1] and % of cells occupied by patches = [0 – 100].

|  | **Low fragmentation** | | **Medium fragmentation** | | **High fragmentation** | |
| --- | --- | --- | --- | --- | --- | --- |
| **Fragmentation index** | **Mean** | **Range (min-max)** | **Mean** | **Range (min-max)** | **Mean** | **Range (min-max)** |
| aggregation index | 85.0 | [76.4 ; 92.1] | 59.4 | [51.8 ; 67.8] | 36.4 | [18.9 ; 49.2] |
| patch cohesion index | 97.9 | [91.0 ; 99.5] | 81.2 | [69.4 ; 91.2] | 48.2 | [23.7 ; 64.9] |
| division index | 0.703 | [0.399 ; 0.970] | 0.992 | [0.974 ; 0.998] | 1.00 | [0.999 ; 1.00] |
| % of cells occupied by patches | 0.619 | [0.319 ; 0.800] | 0.310 | [0.234 ; 0.422] | 0.067 | [0.033 ; 0.104] |

**Table S3.** Scale parameter estimates ($\hat{\alpha}$) of network degree distribution from the fit of a linear model to the log-transformation of degree frequency against the log-transformation of degree. Power function is like $P\left( X=k \right)\propto k^{-\alpha}$ with $\alpha$ being the scale parameter and $k$ the degree. One model was fitted for each sampling frequency and the range (minimum-maximum) of $\hat{\alpha}$ is reported here together with the range of adjusted R^2^ of the linear models for each type of landcape fragmentation and each simulated movement process: F: Foraging, F+Pe: Foraging and avoidance of an elusive predator, F+Ps: Foraging and avoidance of a stalking predator, F+T: Foraging and territoriality.

|  | | **F** | | **F + Pe** | | **F + Ps** | | **F + T** | |
| --- | --- | --- | --- | --- | --- | --- | --- | --- | --- |
|  | $\hat{\boldsymbol{\alpha}}$ | | **R^2^** | $\hat{\boldsymbol{\alpha}}$ | **R^2^** | $\hat{\boldsymbol{\alpha}}$ | **R^2^** | $\hat{\boldsymbol{\alpha}}$ | **R^2^** |
| Low fragmentation | [1.92; 2.66] | | [0.82 ; 0.95] | [1.86 ; 2.38] | [0.86 ; 0.96] | [1.94 ; 2.46] | [0.87 ; 0.97] | [2.08 ; 2.90] | [0.64 ; 1.00] |
| Medium fragmentation | [1.51 ; 2.11] | | [0.74 ; 0.86] | [1.75 ; 2.21] | [0.75 ; 0.84] | [1.81 ; 2.35] | [0.71 ; 0.83] | [1.65 ; 2.37] | [0.72 ; 0.83] |
| High fragmentation | [1.59 ; 2.16] | | [0.63 ; 0.77] | [1.64 ; 2.73] | [0.61 ; 0.79] | [1.82 ; 2.84] | [0.66 ; 0.84] | [1.80 ; 2.60] | [0.65 ; 0.79] |

**Table S4.** Selection coefficients (log(OR)) with their 95% confidence interval (95% CI) and associated p-value for fixed effects of mixed-effects resource selection function of 10 ibex in June 2017, in the Belledonne massif in France.

| Characteristic | log(OR)^1^ | 95% CI | p-value |
| --- | --- | --- | --- |
| **Land cover class** |  |  |  |
| *Grassland* | — | — |  |
| *Closed forest* | 0.137 | -0.065, 0.338 | 0.2 |
| *Herbaceous - mineral* | 0.024 | -0.098, 0.145 | 0.7 |
| *Low ligneous* | 0.181 | 0.061, 0.300 | **0.003** |
| *Mineral* | -0.176 | -0.305, -0.047 | **0.008** |
| *Open forest* | -0.191 | -0.500, 0.117 | 0.2 |
| *Other* | 0.061 | -0.138, 0.259 | 0.5 |
| *Snow* | -1.22 | -1.64, -0.798 | **<0.001** |
| **Northness** | 0.023 | -0.029, 0.076 | 0.4 |
| **Dist. to steep slope (log)** | -0.075 | -0.095, -0.054 | **<0.001** |
| **Random effect (**$\boldsymbol{\sigma}^{\boldsymbol{2}}\boldsymbol{[SE]}$**)** |  | | |
| Individual ID | 0 [0] | | |
| **k-fold** **(mean and range of** $\overline{\boldsymbol{r}_{\boldsymbol{s}}}$**)**  Observed locations  Random locations | 0.82 [0.55 – 0.95]  0.00 [-0.90 – 0.81] | | |
| ^1^OR = Odds Ratio | | | |

| 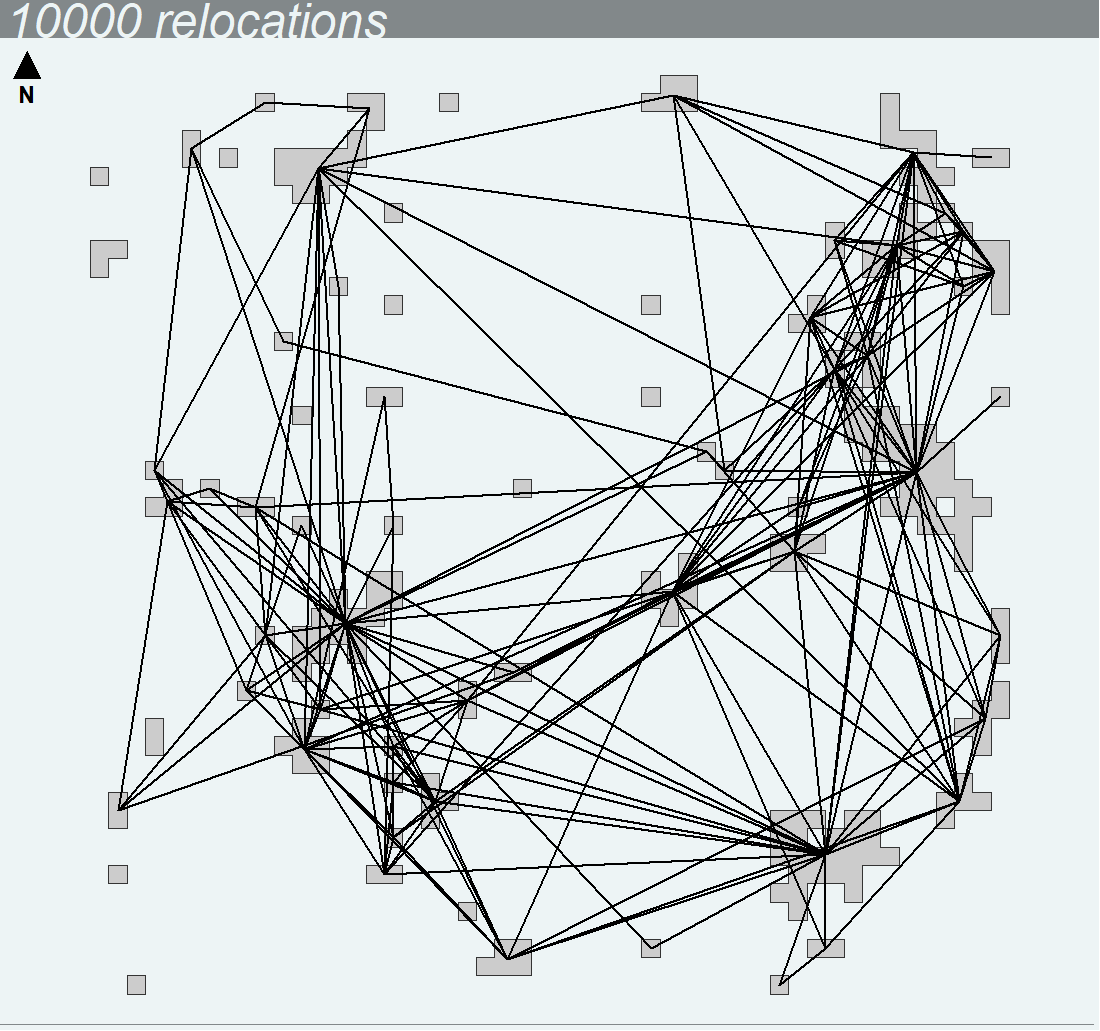 | 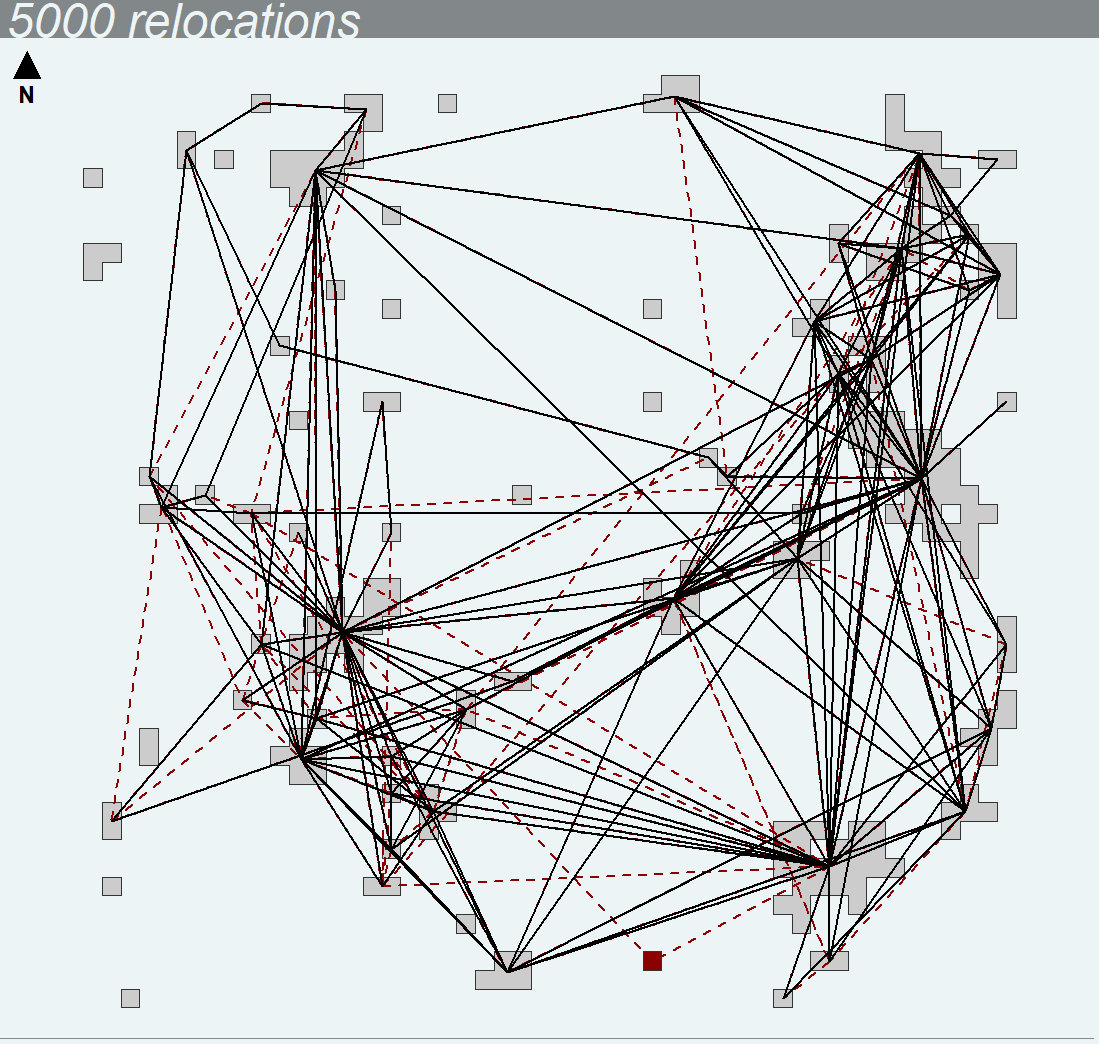 |
| --- | --- |
| 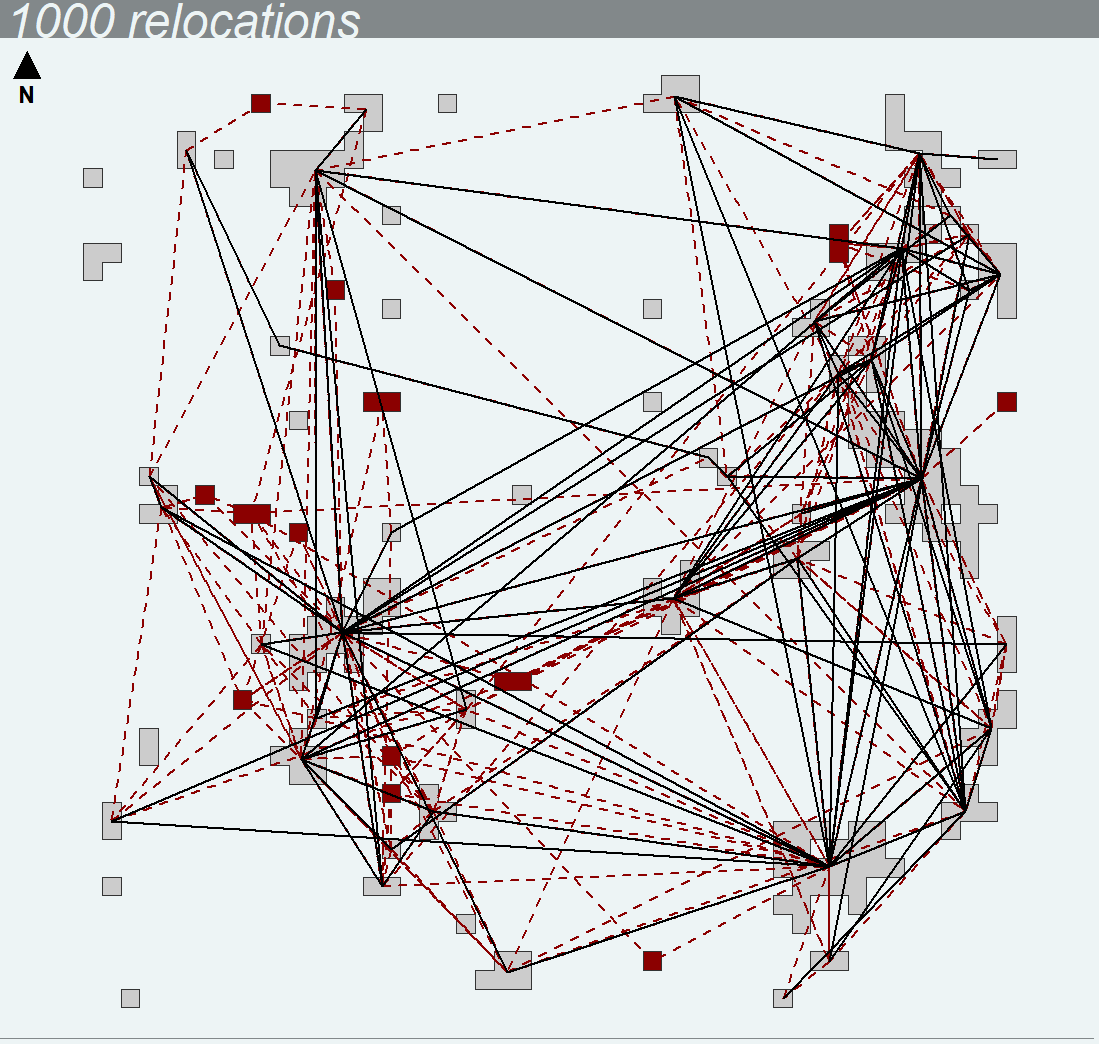 | 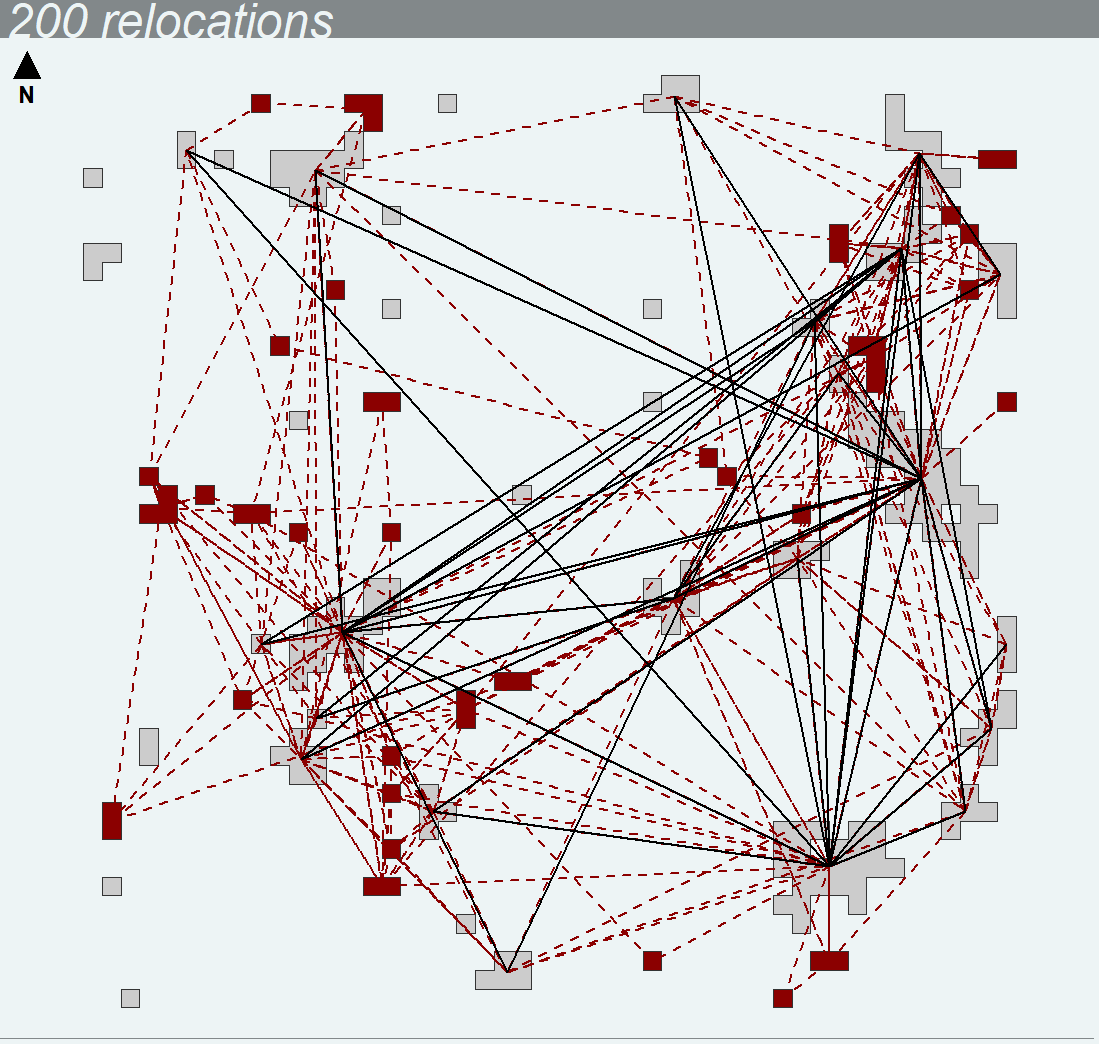 |

Undetected patch

Undetected link

**Figure S1.** Spatial network from individual trajectory of a forager moving among ressource patches (i.e., movement process F) in a highly fragmented landscape. The networks have been built with the same individual trajectory but with different sampling frequency: either 10 000 relocations (full trajectory), 5000 relocations (1 location out of 2), 1000 relocations (1 location out of 10) and 200 relocations (1 location out of 50). Red patches and red dotted links are patches and links, respectively, that are detected from the full trajectory but not with the resampled trajectory.

| 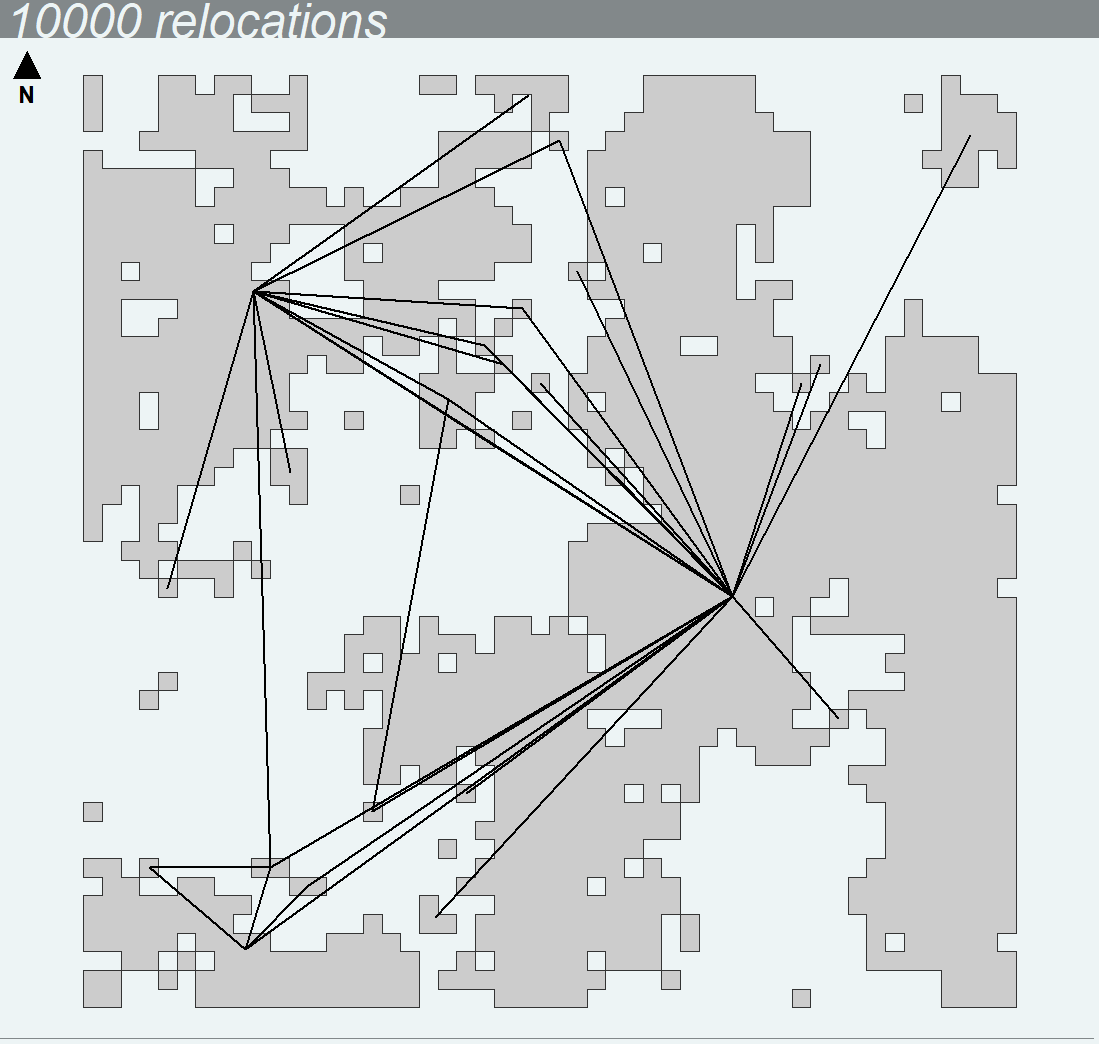 | 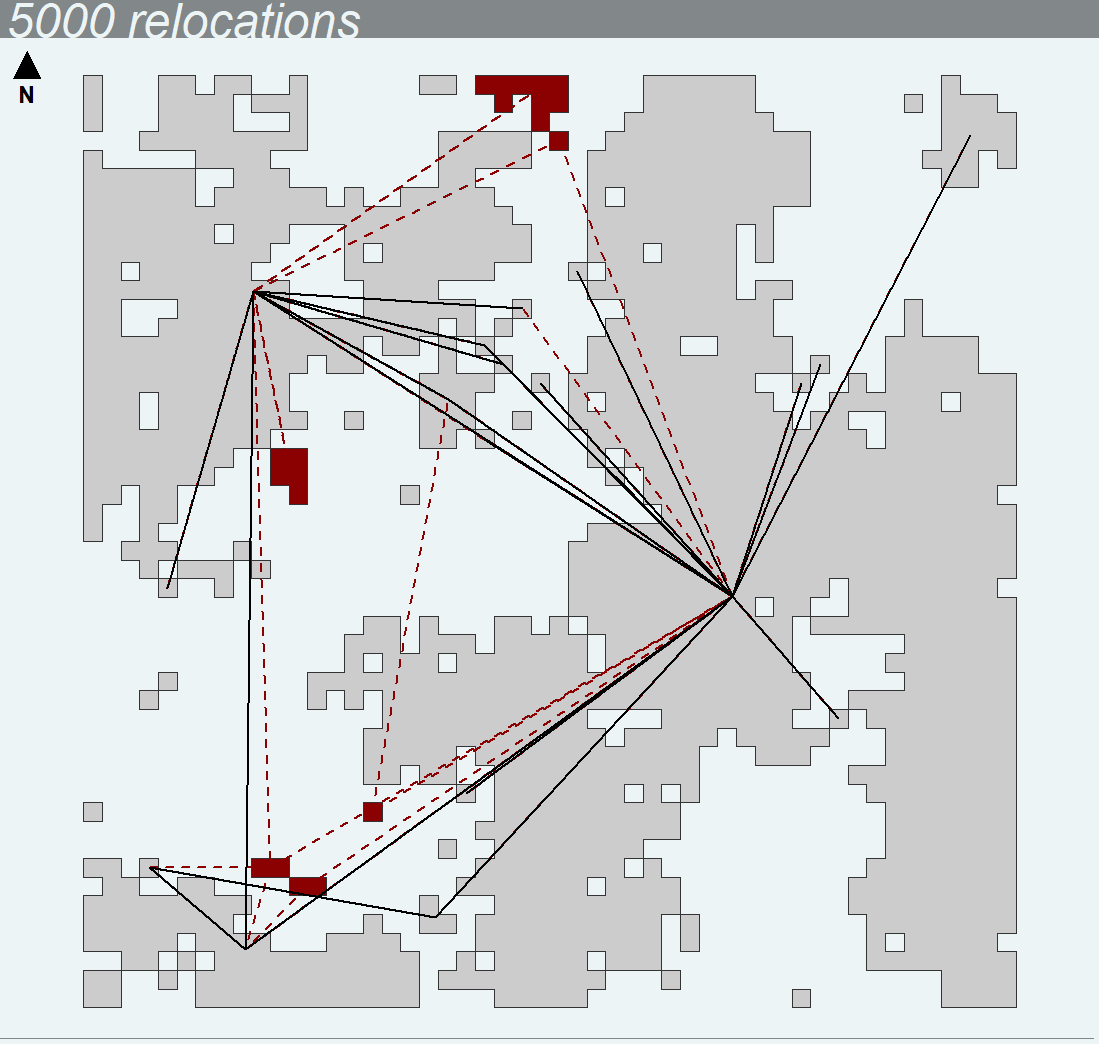 |
| --- | --- |
| 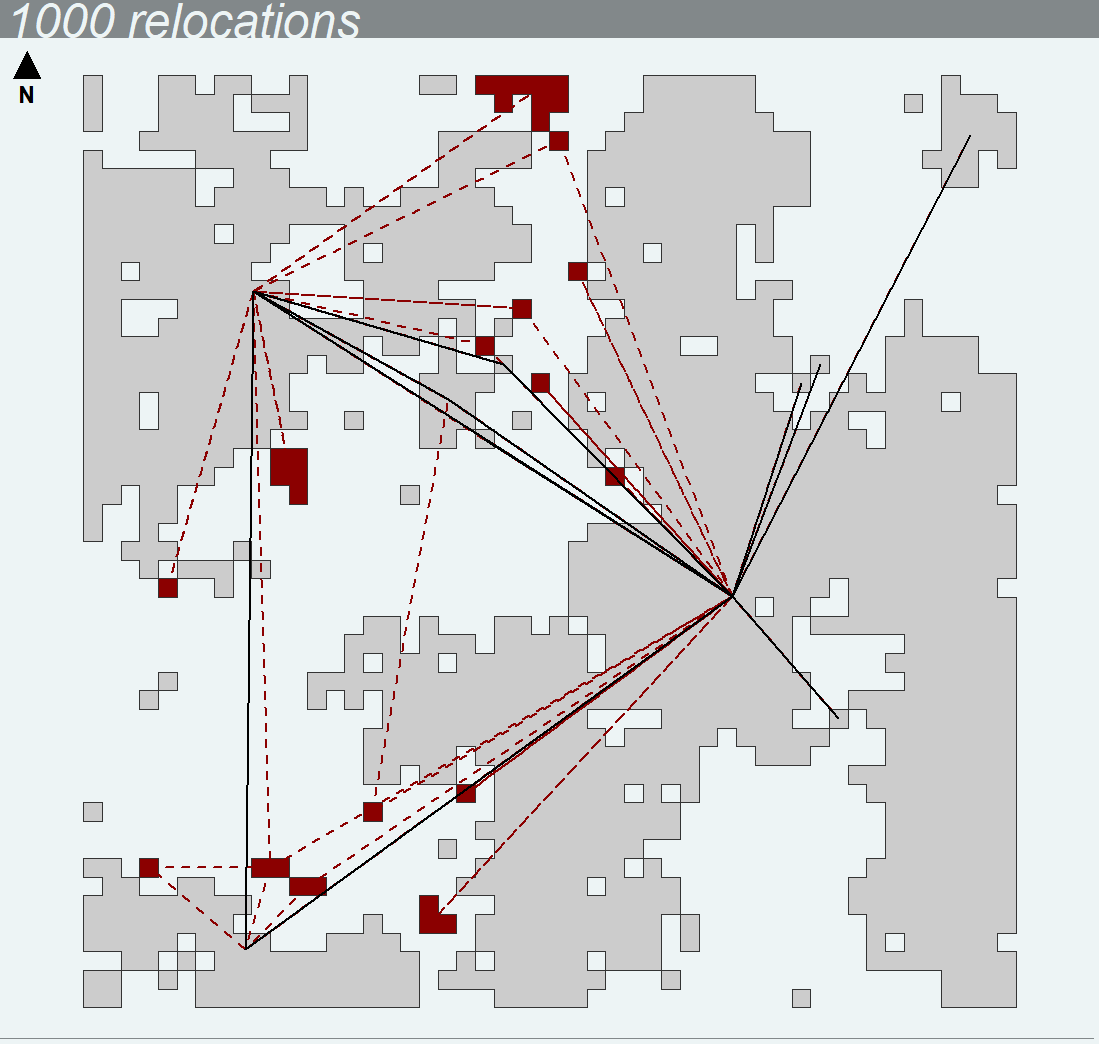 | 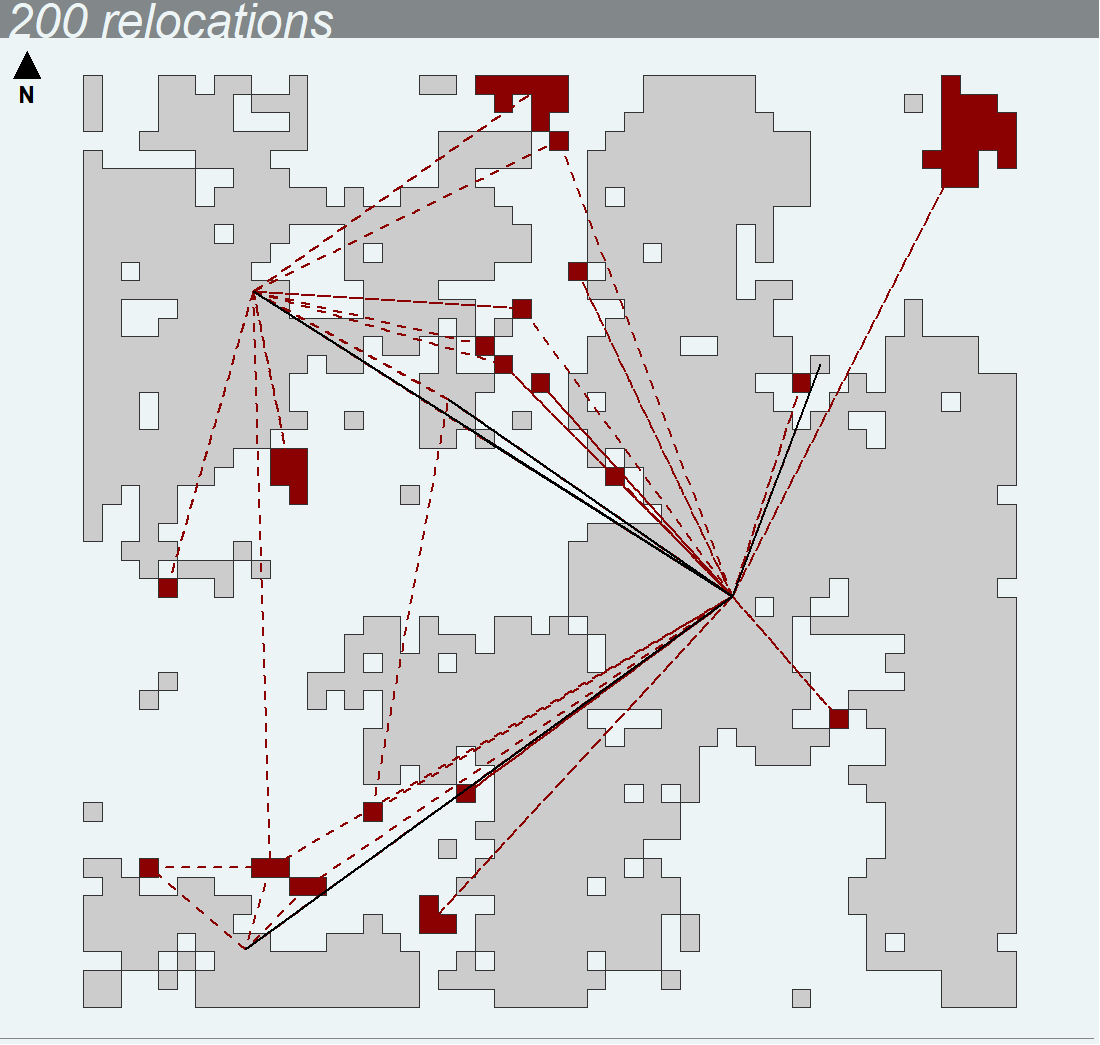 |

Undetected patch

Undetected link

**Figure S2.** Spatial network from individual trajectory of a forager moving among ressource patches (i.e., movement process F) in a low fragmented landscape. The networks have been built with the same individual trajectory but with different sampling frequency: either 10 000 relocations (full trajectory), 5000 relocations (1 location out of 2), 1000 relocations (1 location out of 10) and 200 relocations (1 location out of 50). Red patches and red dotted links are patches and links, respectively, that are detected from the full trajectory but not with the resampled trajectory.


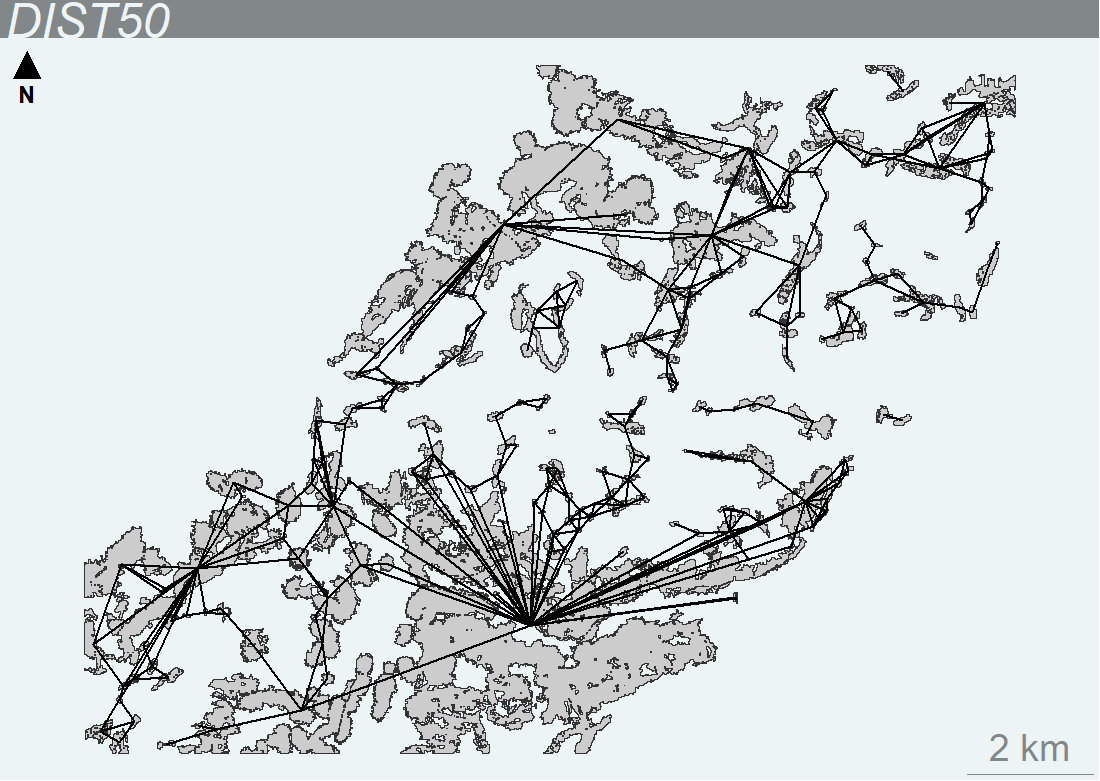

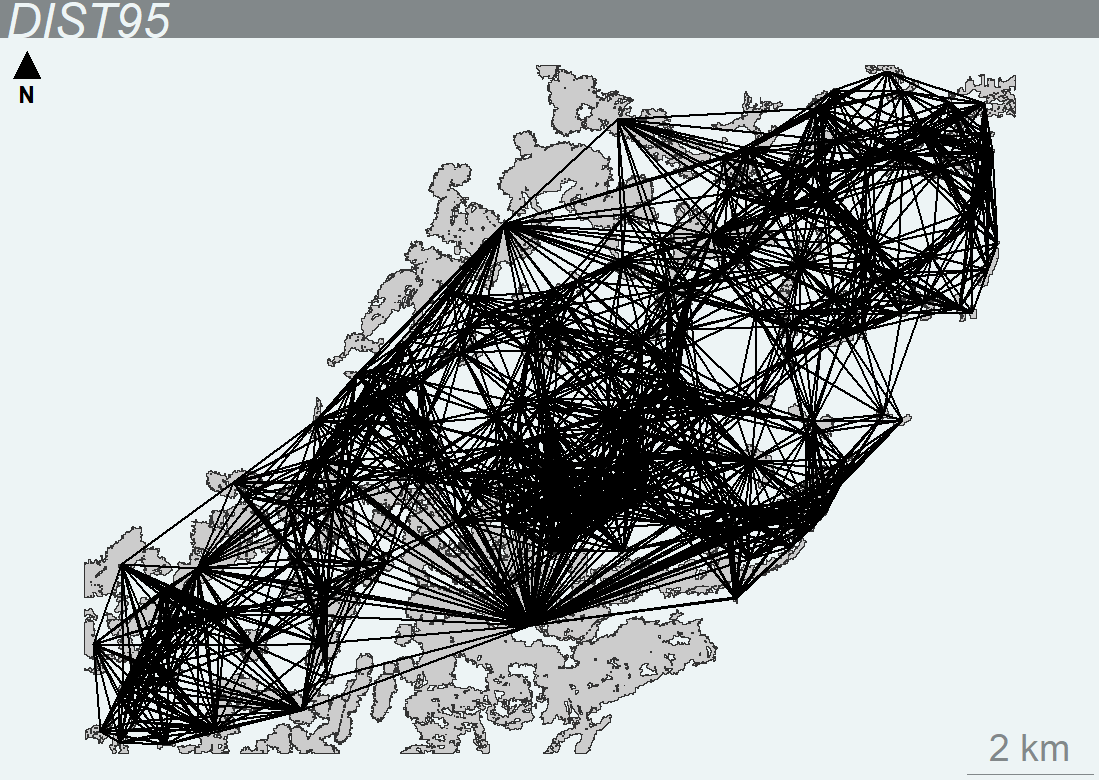

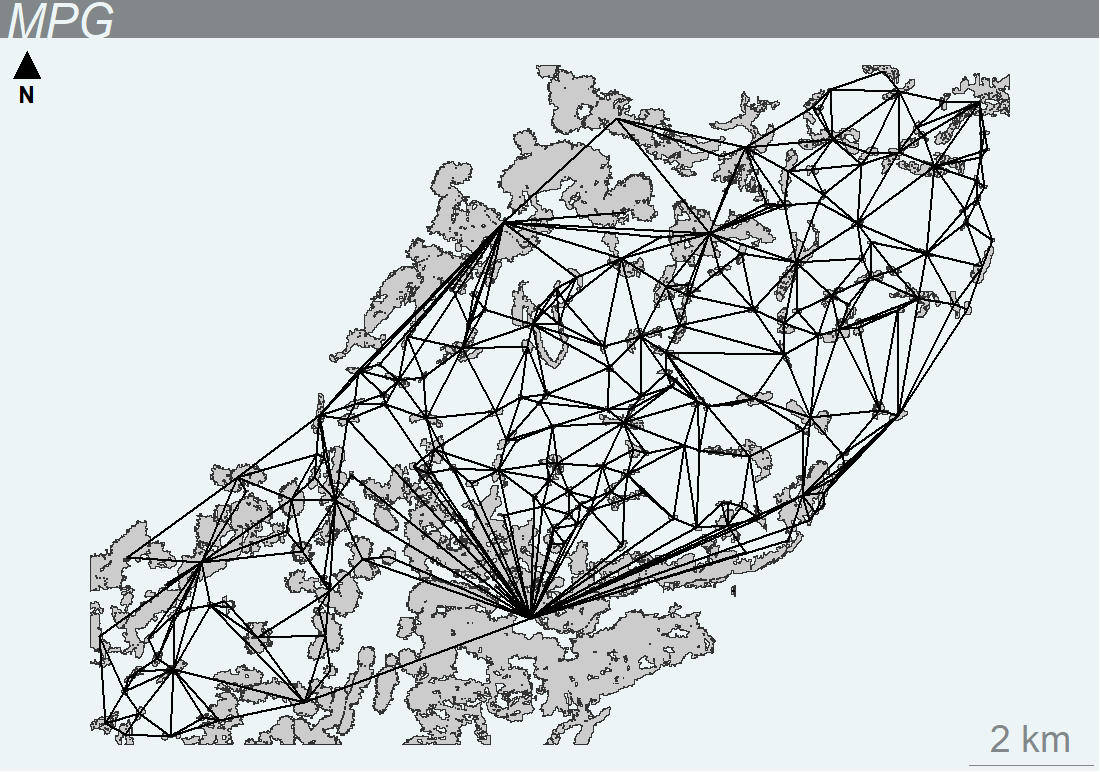


**Figure S3**. Prediction of spatial networks for Alpine ibex in french Belledonne massif in June 2017 from the DIST50, the DIST95 and the MPG. DIST50: distance-based network where patches closer than *L_50%_* are connected, DIST95: distance-based network where patches closer than *L_95%_* are connected, MPG: minimum planar graph. *L_50%_* is the median length of realized links obtained from unique inter-patch moves of the complete simulated trajectory and *L_95%_* is the 95% quantile of the distribution of realized link length.
